# MultiDentNet: a unified deep learning framework for multi-class dental condition screening and preliminary oral lesion triage

**DOI:** 10.1038/s41598-026-52264-6

**Published:** 2026-05-13

**Authors:** Ali Zeydi Abdian, Mohammad Masoud Javidi, Najme Mansouri, Farzaneh Mehranfar, Sahar Cheperli

**Affiliations:** 1 Department of Computer Science, Shahid Bahonar University of Kerman, Kerman, Iran; 2 Faculty of Shahid Bahonar University of Kerman, Kerman, Iran; 3 Department of Periodontics, School of Dentistry, Ahvaz Jundishapur University of Medical Sciences, Ahvaz, Iran; 4 Private Practice, Periodontist and Implantologist, Tehran, Iran

**Keywords:** Dental diagnostics, Oral lesion screening, Deep learning ensemble, Graph convolutional networks, Class imbalance, Triage support, Interpretability, Cancer, Computational biology and bioinformatics, Diseases, Health care, Medical research

## Abstract

To develop and evaluate MultiDentNet, a unified deep learning framework for multi-class dental condition screening and preliminary risk stratification of cancer-suspicious oral lesions, utilizing backbone-diverse ensembling and inter-class relational modeling. Four complementary CNN backbones (DenseNet-121, EfficientNetV2-S, ResNet-50, Inception-V3) integrated with Squeeze-and-Excitation (SE) attention, graph convolutional networks (GCNs), and multi-task learning were fused via validation-optimized weighting. The framework was evaluated on 10,235 clinical intraoral images (five conditions) and 940 clinically labeled oral lesion images (cancer-suspicious vs. non-cancer-suspicious; histopathological confirmation unavailable). Performance was assessed using accuracy, Cohen’s $\kappa$, Matthews correlation coefficient (MCC), false negative rate (FNR), and bootstrapped 95% confidence intervals (CIs), alongside simulated domain-shift robustness testing. The ensemble achieved 99.70% accuracy (95% CI 99.32–100.00%) for dental classification ($\kappa = 0.996$) and 95.71% (95% CI 92.20–98.58%) for oral lesion risk stratification ($\kappa = 0.913$). For cancer-suspicious lesions, recall reached 97.31% (FNR: 2.69%, 95% CI 0.00–7.15%). Architectural diversity successfully mitigated class imbalance, significantly reducing the Hypodontia FNR to 1.65% (95% CI 0.00–4.24%) compared to single-model baselines. Performance demonstrated moderate resilience to acquisition variability ($\Delta$accuracy $\approx$ 8–12% degradation under brightness and contrast perturbations) but degraded substantially under high-frequency noise. Grad-CAM visualizations localized attention to clinically relevant morphological features. MultiDentNet provides an interpretable, efficient baseline for dental screening and lesion triage. Serving as an adjunctive proof-of-concept, its metrics reflect an upper bound due to reliance on single-center, clinically labeled data. Prospective multi-center validation with histopathological standards remains necessary. Code: https://github.com/Aliyar4061/MultiDentNetV3. Positioned as an adjunctive triage tool for resource-constrained settings, high-volume workflows, and tele-dentistry, designed to augment clinician-in-the-loop oversight.

## Introduction

Oral health is a fundamental component of overall systemic well-being. Highly prevalent conditions such as dental caries, gingivitis, and oral mucosal lesions affect billions of individuals worldwide, substantially impairing quality of life and contributing to systemic health complications^1,2^. Early and accurate detection of these conditions is essential to prevent disease progression and associated morbidity. In routine clinical practice, diagnosis predominantly relies on visual–tactile examination and radiographic assessment. While clinically indispensable, these methods are inherently subjective, time-consuming, and susceptible to inter-observer variability, particularly in early-stage or visually ambiguous presentations^3^.

These diagnostic challenges are compounded by a global shortage of dental professionals, which limits access to timely and specialized oral healthcare in many regions^4^. Consequently, there is a pressing need for automated, objective, and scalable diagnostic support systems that can assist clinicians and enable population-level screening. Beyond clinical efficiency, this need carries important implications for health equity, as oral diseases impose substantial socioeconomic burdens through treatment costs, productivity losses, and reduced quality of life, with disproportionately greater impacts on resource-limited communities lacking access to specialized care^4^.

Beyond workforce shortages, a particularly critical challenge lies in the early identification of oral lesions with malignant potential. Oral potentially malignant disorders (OPMDs) represent a heterogeneous group of conditions associated with an elevated risk of progression to oral cancer. The absence of standardized and widely accessible screening protocols for these lesions constitutes a significant public health concern, as early detection is strongly associated with improved clinical management and patient outcomes^5^. Developing tools that support rapid, accurate, and accessible assessment of both common dental conditions and potentially malignant lesions is therefore a clear clinical priority.

Recent advances in artificial intelligence, particularly deep learning, have demonstrated promising performance in automated analysis of dental and intraoral images^6–10^. These studies highlight the potential of AI-based systems to assist clinicians in disease detection, risk stratification, and clinical decision-making for population-level screening. However, most existing approaches focus on isolated diagnostic tasks or a narrow subset of conditions. In real-world screening scenarios, clinicians are required to identify a dominant condition while simultaneously recognizing lesions that may require specialist referral. Addressing this practical need motivates the development of unified frameworks capable of supporting image-level screening across multiple dental conditions, while providing auxiliary assessment of lesion risk.

In this study, we propose MultiDentNet, a deep learning framework designed for population-level dental screening. The framework is formulated to identify a single dominant dental condition per image, while providing an auxiliary binary assessment to flag lesions requiring specialist referral. By prioritizing image-level diagnostic efficiency and interpretability, the proposed framework aligns with practical screening workflows. Detailed methodology, comparisons with prior work, and justification of research gaps are provided in later sections.

The remainder of this paper is organized as follows. Section Related works reviews related work on deep learning-based dental and oral lesion analysis. Section Datasets describes the datasets, annotation strategy, preprocessing steps, and problem formulation. Section Methodology presents the proposed MultiDentNet framework and learning strategy. Section Evaluation metrics outlines the evaluation metrics and experimental setup. Section Results reports the experimental results, followed by discussion and clinical implications in Section Discussion. Finally, Section Conclusions concludes the paper and highlights future research directions. Supplementary materials contain visualizations (Supplementary Figs. S1–S8).

## Related works

Deep learning (DL) has become a central paradigm in dental image analysis, progressively evolving from task-specific models toward broader screening-oriented frameworks. This section reviews representative studies in dental diagnostics, categorizing them according to diagnostic scope and methodological paradigm, and identifies persisting limitations that motivate the proposed approach.

### Single-task dental diagnostic models

Early DL-based research in dentistry primarily focused on detecting or classifying individual conditions. Dental caries detection has been extensively studied using both segmentation and classification paradigms. U-Net-based architectures have demonstrated strong performance in delineating carious lesions in radiographic images due to their encoder-decoder structure and skip connections^11^. Classification-oriented approaches have employed various deep architectures to improve feature representation for caries detection^2,6^. For periodontal disease assessment, transfer learning with pre-trained convolutional backbones has shown promising results in grading disease severity from dental radiographs^12^.

Beyond inflammatory conditions, DL techniques have been applied to hypodontia detection using attention-guided CNNs to enhance interpretability^1^, automated tooth identification and numbering in panoramic images^13^, mandibular landmark localization^14^, and lymph node texture-based metastasis prediction in oral cancer^15^. Although these approaches often achieve high accuracy within narrowly defined tasks, their single-condition focus limits their suitability for comprehensive screening, as multiple independent systems are required to cover the diverse diagnostic categories encountered in routine clinical practice.

### Multi-condition and multi-class approaches

To expand diagnostic coverage, recent studies have explored models capable of handling multiple dental conditions within a unified framework. These approaches can be broadly categorized into two groups.

**Multi-condition segmentation frameworks**: aim to localize different pathologies at the pixel level. A representative example is the work of Liu et al.^8^, who proposed a deep segmentation framework for intraoral images to simultaneously delineate dental calculus, gingivitis, and caries. While such methods provide fine-grained spatial information that is valuable for specialist-driven assessment, they require labor-intensive pixel-level annotations, incur higher computational costs, and are typically limited to a small subset of closely related conditions. Moreover, their primary objective is lesion localization rather than rapid image-level screening or clinical triage.

**Multi-class image-level classification models**: assign a single diagnostic label per image from a predefined set of conditions. Studies such as Hsieh et al.^16^ (employing multimodal feature fusion) and Makarim et al.^17^ demonstrate broad diagnostic coverage at the image level with substantially lower annotation requirements compared to segmentation approaches. However, standard multi-class formulations do not explicitly model inter-condition clinical relationships or provide integrated risk stratification for high-consequence pathologies such as potentially malignant oral lesions. Furthermore, these frameworks often struggle with severe class imbalance for rare conditions and lack mechanisms for clinical interpretability.

In contrast to both paradigms, the present work adopts a single-label multi-class formulation aligned with population-level screening workflows, where a dominant condition is identified per image to guide clinical decision-making, while a dedicated pathway enables preliminary risk stratification of oral lesions as an auxiliary binary output.

### Deployment-oriented advances

Several auxiliary research directions have focused on improving the deployability of dental AI systems. Lightweight network designs, including MobileNet-based architectures, pruning strategies, and efficient convolutional operations, have been proposed to enable real-time inference in resource-constrained environments^18,19^. Explainable AI (XAI) techniques such as Grad-CAM and attention visualization have increasingly been incorporated to improve transparency and clinician trust^1,20^. In addition, advanced data augmentation, domain adaptation, and semi-supervised learning strategies have been explored to mitigate limited data availability and enhance robustness to image variability^21^.

### Identified research gaps and technical innovations

A critical analysis of the current literature reveals five fundamental limitations that motivate the design of MultiDentNet. Addressing these gaps is essential for the transition of dental AI from isolated tasks to robust, context-aware clinical deployment:

- *Fragmented single-task focus.* Most existing frameworks address isolated conditions through dedicated single-task models (e.g., caries-only detection^11^ or periodontitis grading^12^). Even the few multi-condition attempts remain restricted to narrow inflammatory subsets (e.g., calculus, gingivitis, and caries^8^) and do not offer a clinically unified pipeline that assigns a single dominant diagnosis across a broad spectrum of dental pathologies, a requirement for rapid triage in screening settings.
- *Absence of oral lesion risk stratification.* Approaches such as Liu et al.^8^ cover exclusively inflammatory dental diseases and entirely omit preliminary stratification of potentially malignant oral lesions. Given the high variability of AI performance in lesion assessment (e.g., 25–96% accuracy^22^), this represents a critical gap for any comprehensive population-level screening programme.
- *Inadequate class imbalance handling.* Standard loss functions bias predictions toward prevalent conditions (e.g., caries), reducing sensitivity for underrepresented pathologies such as hypodontia in single-label multi-class settings^9^. Furthermore, vision-based dental AI often exhibits an “optimistic bias,” overestimating confidence on minority classes without proper threshold calibration^23^.
- *Ignored inter-condition relationships.* Despite well-established clinical correlations (for instance, the frequent co-occurrence of caries and gingivitis^10^), most models treat dental conditions as independent, thereby discarding valuable relational priors that could improve diagnostic reasoning.
- *Suboptimal feature integration and limited robustness.* Ensemble methods often rely on naive fusion strategies without adaptive recalibration^19^, while many existing models lack comprehensive augmentation, leaving them vulnerable to the high intra‑oral image variability encountered across devices and illumination conditions^18^.

To address these gaps within a clinically practical, single-label multi-class screening paradigm, MultiDentNet introduces the following innovations:

- *Hybrid CNN-GCN ensemble architecture.* Four complementary pre-trained CNN backbones (DenseNet-121, EfficientNetV2-S, ResNet-50, Inception-V3) are each equipped with Squeeze-and-Excitation attention, multi-task heads, and a lightweight Graph Convolutional Network that explicitly models inter-condition relationships through a learnable adjacency matrix. The four branches are trained independently and combined at the logit level via validation-accuracy-weighted averaging, preserving single-label output semantics while benefiting from architectural diversity.
- *Single-label multi-class formulation with auxiliary risk screening.* The framework assigns one primary diagnosis per image from five common dental conditions (caries, gingivitis, discoloration, ulcers, hypodontia) to mirror routine triage workflows. In parallel, a dedicated binary pathway, trained on a separate dataset of clinically labeled oral lesions, performs preliminary “cancer-suspicious” versus “non-cancer-suspicious” stratification. This auxiliary output is explicitly framed as a screening hint requiring histopathological confirmation, and it does not influence the primary dental condition decision.
- *Squeeze-and-Excitation attention for channel-wise recalibration.* Integrated into every backbone, SE blocks adaptively emphasise diagnostically informative feature maps, improving the quality of the individual models and, consequently, the ensemble consensus.
- *Imbalance-aware loss formulation.* The combination of focal loss with label smoothing counteracts the dominance of majority classes and has been specifically designed to boost sensitivity for rare conditions such as hypodontia.
- *Comprehensive data augmentation and test-time augmentation.* Aggressive geometric, photometric, and elastic transformations during training, together with inference-time augmentation (flips, rotations), harden the model against real-world imaging variability without requiring additional data.
- *Validation-optimised ensemble fusion.* Logit-level aggregation weights are derived from validation accuracy, allowing the ensemble to capitalise on the complementary inductive biases of its members while suppressing outlier predictions.
- *Integrated interpretability suite.* Clinically oriented insights are provided through GCN-derived inter-condition affinity visualisations, SE attention maps, and gradient-based saliency (Grad-CAM). All interpretability outputs are presented as supportive tools that highlight model attention patterns rather than as standalone clinical evidence.
- *Proof-of-concept oral lesion risk stratification.* Evaluation on a 940-image, publicly available dataset of lip and tongue lesions demonstrates the feasibility of binary risk assessment as an auxiliary output. Consistent with the need for rigorous real-world validation, results are strictly reported as preliminary screening capability, and clinical translation would require prospective, multi-centre validation with histopathologically verified ground truth.

These innovations collectively position MultiDentNet as a unified framework for single-label multi-class dental screening with integrated oral lesion triage, specifically designed for population-level settings where rapid identification of a dominant condition can effectively guide referral decisions. Detailed architectural descriptions are provided in Section Methodology.

Table 1 provides a structured comparison of representative deep learning studies in dental image analysis, highlighting scope, architectures, conditions addressed, strengths, and limitations.

**Table 1:** Comparative analysis of deep learning approaches in dental image analysis.

Study (References)	Scope	Key architecture	Conditions addressed	Key strength	Primary limitation
Lian et al.^6^	Single-Task	DenseNet/ResNet (Classification)	Caries	High accuracy for specific task	Single-condition focus; cannot screen multiple diseases
Bayrakdar et al.^11^	Single-Task	U-Net (Segmentation)	Caries	Precise lesion localisation	Requires pixel-level annotations; limited to one condition
Chen et al.^12^	Single-Task	VGG16 (Classification)	Periodontitis	Effective severity grading	Single-condition focus; no multi-disease analysis
Liu et al.^8^	Multi-Task	Segmentation CNN	Calculus, Gingivitis, Caries	Simultaneous pixel-level localisation of 3 conditions	Limited to inflammatory conditions; no oral lesion triage; costly annotations
Hsieh et al.^16^	Multi-Class	Multimodal Fusion CNN	Multiple conditions including gingivitis	Broad image-level screening with lower annotation burden	Does not model inter-condition relationships; limited rare-condition sensitivity
Makarim et al.^17^	Multi-Class	Efficient CNN	Multiple conditions	Designed for clinical deployment	May trade accuracy for efficiency; lacks interpretability
Lightweight Studies (e.g.,^18,19^)	Auxiliary	MobileNet/Pruning	Varies	Low computational cost; mobile-friendly	Reduced capacity; not a complete diagnostic framework
XAI Studies (e.g.,^1,20^)	Auxiliary	Grad-CAM/Attention	Varies	Improves interpretability	Typically post-hoc; adds computational overhead
MultiDentNet (Ours)	Unified Framework	Hybrid CNN-GCN Ensemble	5 Dental Conditions (single-label) + Oral Lesion Triage (binary auxiliary)	Comprehensive screening with cancer-suspicious triage; interpretable; handles class imbalance	Assumes single dominant diagnosis per image; requires external multi-centre validation on histopathologically confirmed data

## Datasets

### Dental condition dataset

The Dental Condition Dataset comprises 9,439 anonymized intraoral images collected from five dental hospitals in diverse regions, along with ethically sourced public repositories, ensuring wide clinical and demographic variability. The dataset is publicly available (see the ’Availability of data and materials’ section in Declarations).

Images are categorized into five clinically relevant conditions:

1. *Caries*: Cavitated or non-cavitated carious lesions.
2. *Gingivitis*: Gingival inflammation (erythema, edema, or bleeding on probing).
3. *Tooth Discoloration*: Extrinsic or intrinsic staining unrelated to pathology.
4. *Ulcers*: Aphthous or traumatic oral ulcers with mucosal disruption.
5. *Hypodontia*: Clinically observed absence of permanent teeth (excluding third molars), requiring radiographic confirmation for definitive diagnosis.

All images were annotated with bounding boxes using a consensus protocol. Images were standardized to 300 DPI with consistent color calibration. Data augmentation (random rotation $\pm 15^\circ$, horizontal flipping, scaling $\pm 10\%$, and controlled noise injection) was applied exclusively to the training set to improve generalization; validation and test sets remained unaugmented.

**Fig. 1:**
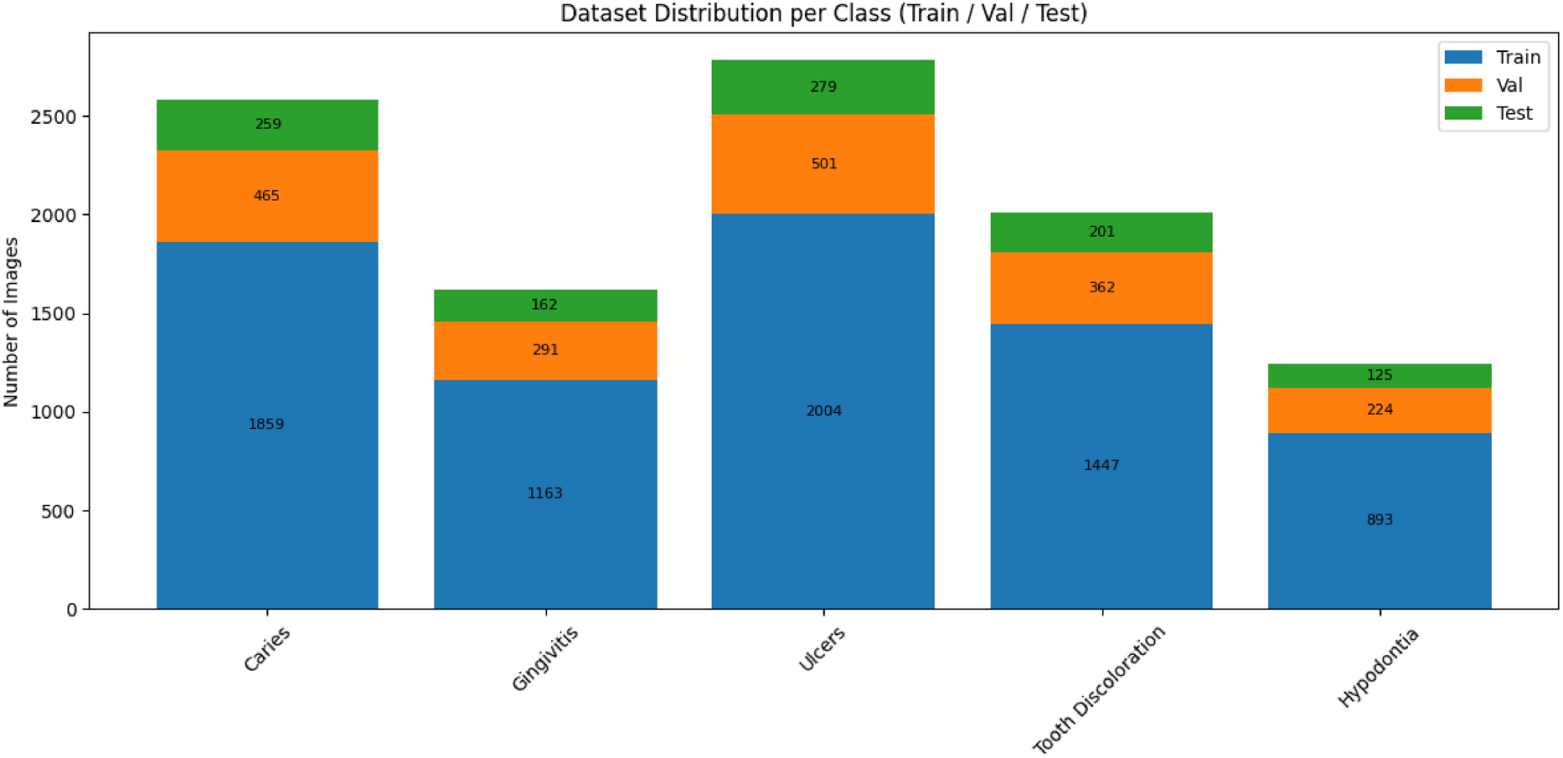
Train/validation/test split configuration for the dental condition dataset.

The dataset was partitioned into training, validation, and test subsets using stratified sampling to preserve the original class distribution across all splits. As shown in Fig. 1, the dental dataset comprises 10,235 images distributed across five clinical conditions, with 7,366 images allocated to training, 1,843 to validation, and 1,026 to testing, corresponding to an approximate 72:18:10 split ratio. This configuration ensures a sufficiently large training set for robust feature learning, a validation set for hyperparameter tuning and model selection, and an independent test set for unbiased evaluation of generalization performance.

Stratification was applied to maintain consistent class proportions across all subsets, thereby reducing sampling bias and improving model stability under class imbalance conditions. Importantly, minority classes such as Hypodontia are preserved in all splits despite their limited overall prevalence, ensuring that the model is exposed to rare but clinically significant conditions during both training and evaluation. Before splitting, exact duplicate images were identified using MD5 hashing and removed. All splits are strictly independent. The duplicate removal script is available in the GitHub repository (https://github.com/Aliyar4061/MultiDentNetV3).

The detailed per-class distribution across training, validation, and test subsets is reported in Table 2, while Fig. 1 provides a visual overview of the dataset allocation.

**Table 2:** Distribution of the dental condition dataset across Training, Validation, and Test subsets.

Condition	Training set	Validation set	Test set	Total
Caries	1,859 (71.97%)	465 (17.99%)	259 (10.03%)	2,583
Gingivitis	1,163 (71.97%)	291 (18.01%)	162 (10.02%)	1,616
Ulcers	2,004 (71.98%)	501 (18.00%)	279 (10.02%)	2,784
Tooth Discoloration	1,447 (71.99%)	362 (18.01%)	201 (10.00%)	2,010
Hypodontia	893 (71.90%)	224 (18.04%)	125 (10.06%)	1,242
Total (Images)	7,366 (71.97%)	1,843 (18.01%)	1,026 (10.02%)	10,235

**Fig. 2:**
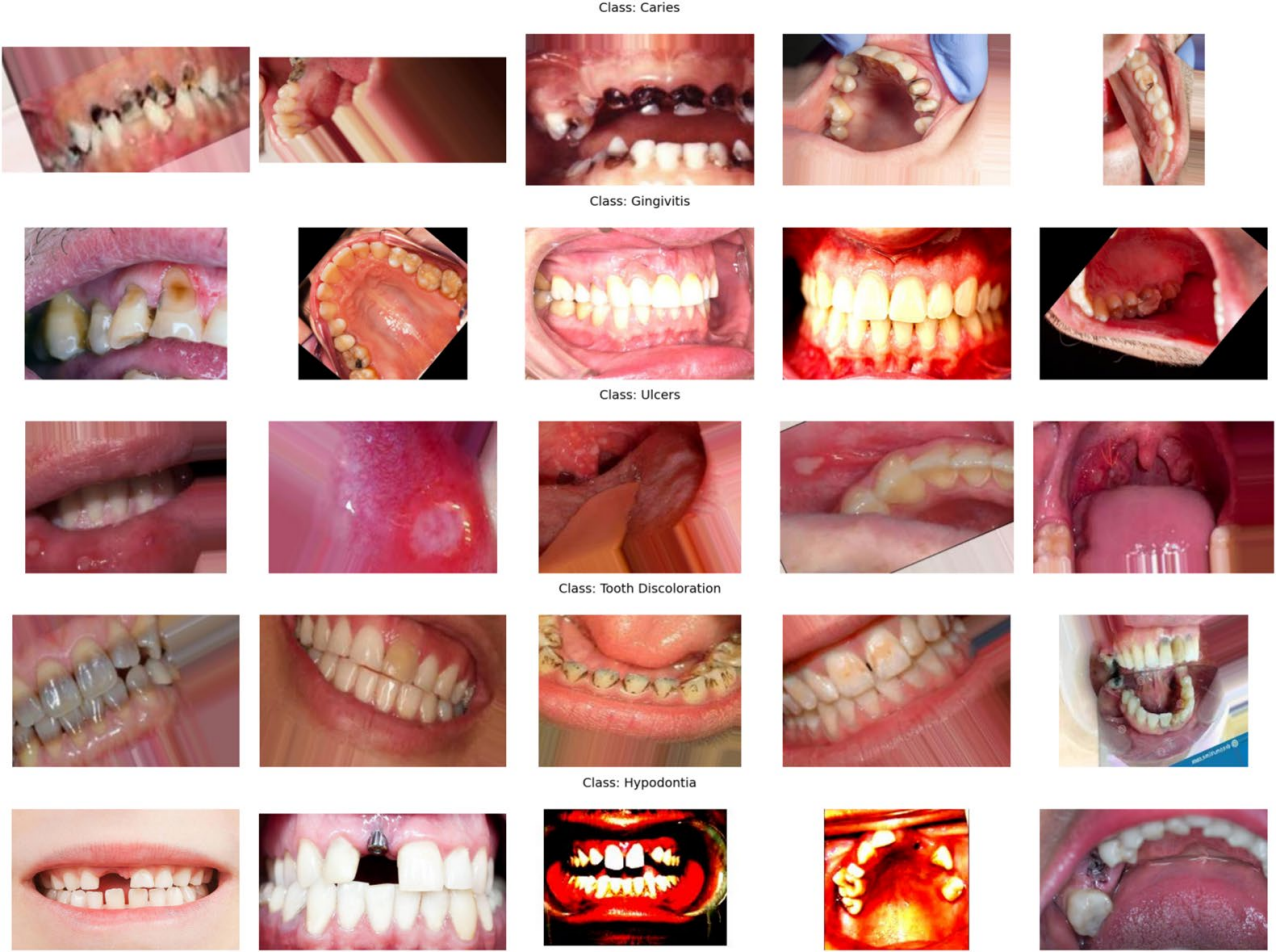
Representative samples from the dental condition dataset.

Hypodontia is underrepresented due to its lower clinical prevalence. Clinical photographs alone cannot reliably distinguish congenital absence from delayed eruption, especially in pediatric or mixed-dentition patients. Consequently, model predictions for this class should be interpreted as visible tooth absence requiring expert validation, ideally supplemented by radiographic confirmation. MultiDentNet is designed strictly as a preliminary screening tool; all positive detections should be referred to dental professionals for comprehensive evaluation, including clinical history, panoramic radiographs, and developmental assessment. Future work should focus on constructing datasets with age-stratified annotations and radiographic labels to enable safer and more accurate modeling. Representative samples are shown in Fig. 2.

### Oral cancer dataset

The Oral Lesion Dataset comprises 940 de-identified clinical images of lips and tongues, publicly available (see Data Availability). Images are clinically labeled by oral medicine specialists. For brevity and operational convenience in image annotation and model prediction, we adopt the simplified binary terms cancer and non-cancer in model outputs to represent the original clinical categories:

- *Cancer*: Potentially malignant or clinically suspicious lesions (490 images; originally labeled “Cancer-Suspicious”).
- *Non-Cancer*: Benign lesions or healthy mucosa (450 images; originally labeled “Non-Cancer-Suspicious”).

Images were standardized to 300 DPI with color calibration. Since comprehensive histopathological confirmation is unavailable for all cases (Section Discussion), the labels reflect *clinical suspicion* rather than pathological ground truth. Accordingly, model predictions are explicitly framed as preliminary triage support to assist expert evaluation, not as definitive diagnostic decisions. Data augmentation was applied exclusively to the training set to prevent evaluation bias.

**Fig. 3:**
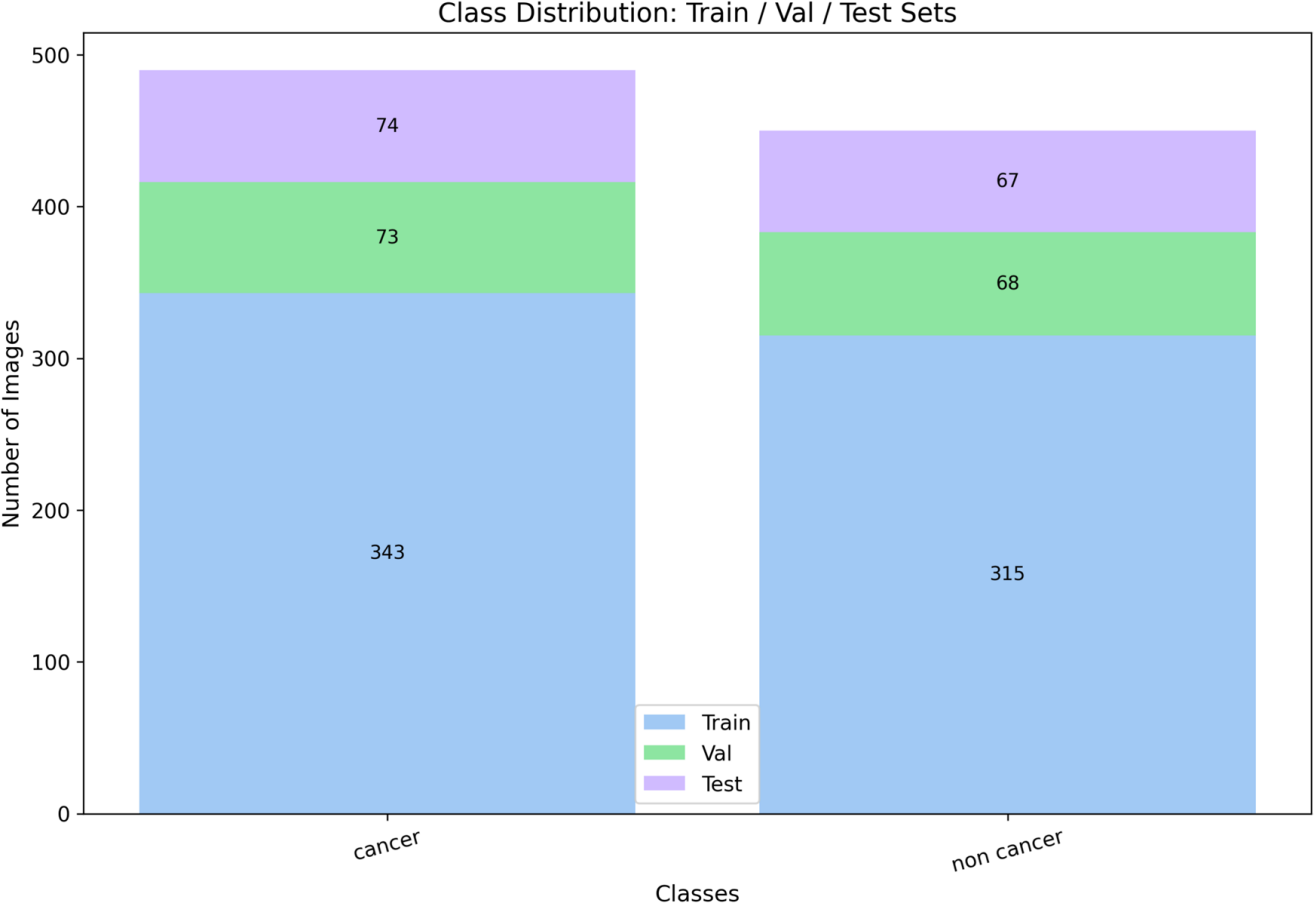
Class distribution across train/validation/test splits.

**Fig. 4:**
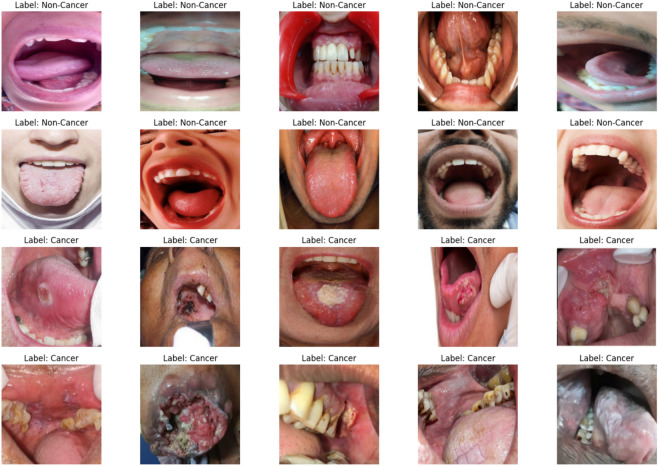
Representative samples: cancer-suspicious vs. non-cancer-suspicious.

Stratified splitting (70:15:15) yielded training (658), validation (141), and test (141) subsets with balanced class representation (Table 3, Fig. 3). Representative samples are shown in Fig. 4.

**Table 3:** Oral lesion dataset distribution.

Class	Train	Val	Test	Total
Cancer-Suspicious	343 (70.0%)	73 (14.9%)	74 (15.1%)	490
Non-Cancer-Suspicious	315 (70.0%)	68 (15.1%)	67 (14.9%)	450
Total	658	141	141	940

#### Annotation protocol and clinical validation

All images were annotated and clinically validated by two board-certified periodontists and implantologists with over 10 years of experience in oral diagnosis and pathology. The annotation process followed a rigorous two-stage protocol to ensure accuracy and reliability:

1. *Independent annotation*: Each expert labeled all images separately, without prior discussion or knowledge of the other’s decisions.
2. *Consensus adjudication*: The annotators subsequently held structured consensus meetings to review every discrepancy and jointly determine the final label for each image. All disagreements were resolved through direct discussion until full agreement was reached.

To ensure high image quality, images exhibiting low resolution (below 300 DPI), poor illumination, motion artifacts, excessive blur, or ambiguous pathology were systematically excluded prior to annotation. Inter-rater agreement was quantified before consensus using Cohen’s $\kappa$ coefficient, which exceeded 0.90 across all diagnostic categories, indicating excellent reliability according to established benchmarks^24^. The final dataset reflects the consensus labels agreed upon by both experts.

#### Dataset preparation and loading

The dental disease dataset was organized into standard train/validation/test splits using stratified random sampling to maintain proportional class distributions across all subsets (72%/18%/10% split ratio). Images were resized to $224\times 224$ pixels using bicubic interpolation and normalized using ImageNet statistics ($\mu = [0.485, 0.456, 0.406]$, $\sigma = [0.229, 0.224, 0.225]$) to ensure compatibility with pretrained backbone architectures. A custom PyTorch Dataset and DataLoader implementation handled efficient batch loading with the following configuration: batch size $B=128$ (adjusted for GPU memory constraints), shuffling enabled for training splits, pinned memory for accelerated GPU transfer, and two worker processes for parallel data loading.

#### Data augmentation and normalization

To enhance model robustness and mitigate overfitting, we implemented an extensive augmentation pipeline during training using the Albumentations library. The complete augmentation strategy comprises:

- **Geometric transformations**:
  - Horizontal flip ($p=0.5$) and vertical flip ($p=0.5$) to account for bilateral symmetry in dental anatomy
  - Random rotation within $\pm 20^\circ$ ($p=0.7$) to simulate varied acquisition angles
  - Elastic deformation ($\alpha =1.0$, $\sigma =50$, $p=0.3$) to model soft-tissue variability
- **Photometric transformations**:
  - Color jittering: brightness, contrast, saturation, and hue perturbations ($\pm 0.1$, $p=0.5$) to simulate illumination variations
  - Contrast Limited Adaptive Histogram Equalization (CLAHE) with clip limit $=2.0$ ($p=0.3$) to enhance local contrast in radiographic images
- **Regularization augmentations**:
  - Coarse dropout with maximum height/width $=0.1\times$ image dimension ($p=0.5$) to prevent feature co-adaptation

For validation and testing phases, only deterministic preprocessing was applied: resizing to $224\times 224$ pixels followed by normalization. During inference, Test-Time Augmentation (TTA) with $K=5$ stochastic transformations (horizontal flip, vertical flip, and rotations at $\pm 15^\circ$) was employed, with final predictions obtained by averaging softmax probabilities across all augmented views to improve prediction stability.

### Problem formulation

Given a preprocessed dental image $\textbf{X} \in \mathbb {R}^{224\times 224\times 3}$, MultiDentNet addresses a single-label multi-class classification task within a unified deep learning framework. The primary objective is to assign exactly one dominant diagnosis from five clinically distinct dental conditions:

1 $$\begin{aligned} \mathcal {C}_{dental} = \{\text {Caries}, \text {Gingivitis}, \text {Hypodontia}, \text {Tooth Discoloration}, \text {Ulcers}\}, \end{aligned}$$

corresponding to label indices $c \in \{0,1,2,3,4\}$. The primary classification objective is formalized as:

2 $$\begin{aligned} \hat{y}_{dental} = \arg \max _{c \in \mathcal {C}_{dental}} P(y_{dental} = c \mid \textbf{X}; \boldsymbol{\theta }), \end{aligned}$$

where $\boldsymbol{\theta } = \{\boldsymbol{\theta }_{\text {backbone}}, \boldsymbol{\theta }_{\text {SE}}, \boldsymbol{\theta }_{\text {GCN}}, \boldsymbol{\theta }_{\text {MTL}}\}$ denotes the complete set of learnable parameters spanning the multi-backbone feature extractors, Squeeze-and-Excitation attention modules, graph convolutional relational layers, and multi-task classification heads.

In parallel, for images routed to the oral lesion screening pathway, the framework performs a preliminary binary risk stratification:

3 $$\begin{aligned} \mathcal {C}_{lesion} = \{\text {Non-Cancer-Suspicious}, \text {Cancer-Suspicious}\}, \end{aligned}$$

optimised via an auxiliary objective to flag potentially malignant conditions requiring immediate review. This dual formulation enables efficient population-level screening where each intraoral image receives a single, clinically actionable primary diagnosis, while severe outlier lesions are prioritized for specialist referral in accordance with clinical triage protocols.

## Methodology

This section presents the comprehensive methodology underlying the MultiDentNet framework, a unified deep learning architecture designed for multi-class classification of dental conditions from medical imaging data. The proposed approach synergistically integrates multiple convolutional neural network (CNN) backbones with channel-wise attention mechanisms, multi-task learning paradigms, graph-based relational modeling, and ensemble aggregation strategies. The methodology encompasses rigorously defined training protocols, advanced regularization techniques, and a statistically robust evaluation framework with uncertainty quantification. All components are mathematically formalized and implementation-ready to ensure reproducibility and facilitate adoption in clinical AI research.

### Model architecture

**Fig. 5:**
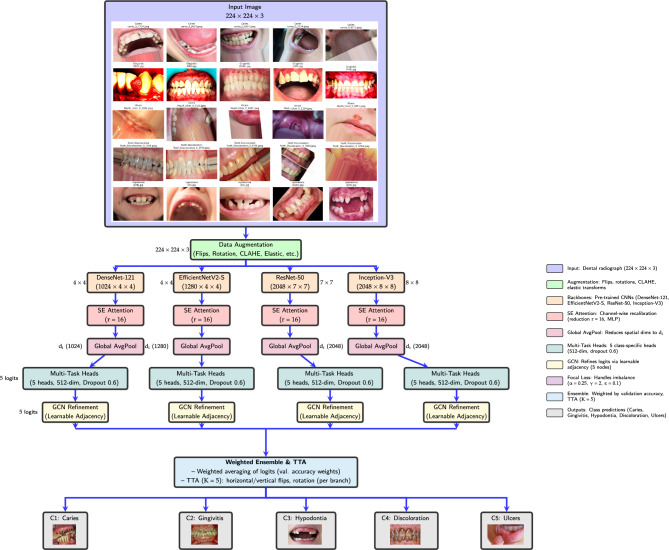
Architecture of the hybrid CNN-GCN framework for dental condition classification. The pipeline integrates four pretrained CNN backbones with SE attention, multi-task heads, GCN-based logit refinement, and weighted ensemble aggregation.

The MultiDentNet architecture, illustrated in Fig. 5, processes input dental radiographs of dimensions $224 \times 224 \times 3$ through a hierarchical pipeline comprising four principal stages: (i) multi-backbone feature extraction, (ii) attention-enhanced feature refinement, (iii) multi-task classification with graph-based relational modeling, and (iv) weighted ensemble aggregation with test-time augmentation. (Note: In the actual implementation, each backbone is trained as an independent model; predictions are fused via weighted ensemble).

#### Multi-backbone feature extraction

To leverage complementary representational capacities, we employ four distinct pretrained CNN architectures as parallel feature extractors: DenseNet-121^25^ (8M parameters), EfficientNetV2-S^26^ (21.5M parameters), ResNet-50^27^ (25.6M parameters), and Inception-V3^28^ (27.2M parameters). Each backbone $\mathcal {B}_i$ processes the augmented input $\textbf{X} \in \mathbb {R}^{224 \times 224 \times 3}$ to produce hierarchical feature maps $\textbf{F}_i \in \mathbb {R}^{d_i \times H' \times W'}$, where $d_i \in \{1024, 1280, 2048\}$ denotes the channel dimension specific to each architecture. In our implementation, each backbone is trained independently; the predictions are subsequently fused via a weighted ensemble.

#### Squeeze-and-excitation attention mechanism

To enhance discriminative feature representation, we integrate Squeeze-and-Excitation (SE) blocks^29^ after each backbone. For feature maps $\textbf{F} \in \mathbb {R}^{C \times H \times W}$, the SE operation computes channel-wise attention weights $\textbf{s} \in \mathbb {R}^C$ via:

4 $$\begin{aligned} \textbf{z} = \frac{1}{H \times W} \sum _{h=1}^{H} \sum _{w=1}^{W} \textbf{F}(:,h,w), \quad \textbf{s} = \sigma (\textbf{W}_2 \delta (\textbf{W}_1 \textbf{z})), \end{aligned}$$

where $\delta$ denotes ReLU activation, $\sigma$ is the sigmoid function, and $\textbf{W}_1 \in \mathbb {R}^{\frac{C}{r} \times C}$, $\textbf{W}_2 \in \mathbb {R}^{C \times \frac{C}{r}}$ are learnable parameters with reduction ratio $r=16$. The recalibrated features are obtained as $\tilde{\textbf{F}} = \textbf{s} \odot \textbf{F}$, where $\odot$ denotes channel-wise multiplication.

#### Multi-task classification heads

Rather than a single shared classifier, we implement five dedicated task-specific heads to enable specialized learning for each dental condition. Each head $\mathcal {H}_c$ ($c \in \{1,\dots ,5\}$) comprises a fully-connected layer (512 units), batch normalization, ReLU activation, dropout ($p=0.6$), and a final linear projection to scalar logit $\ell _c$:

5 $$\begin{aligned} \ell _c = \textbf{w}_c^\top \delta (\textbf{BN}(\textbf{W}_c \textbf{f}) + \textbf{b}_c) + b_c', \end{aligned}$$

where $\textbf{f} \in \mathbb {R}^{d_i}$ is the globally pooled feature vector obtained via adaptive average pooling: $\textbf{f} = \text {GAP}(\tilde{\textbf{F}})$.

#### Graph convolutional relational modeling

To explicitly model inter-class dependencies informed by clinical knowledge and data-driven correlations, we introduce a Graph Convolutional Network (GCN)^30^ layer that refines the logits $\boldsymbol{\ell } = [\ell _1, \dots , \ell _5]^\top$. The adjacency matrix $\textbf{A} \in \mathbb {R}^{5 \times 5}$ is initialized as $\textbf{A} = \textbf{I} + 0.05$ with diagonal dominance, then row-normalized. The GCN propagation rule is:

6 $$\begin{aligned} \boldsymbol{\ell }' = \boldsymbol{\ell } + \text {ReLU}(\textbf{A} \boldsymbol{\ell }), \end{aligned}$$

where $\boldsymbol{\ell }'$ denotes the relationally refined logits. Optionally, $\textbf{A}$ can be made learnable via parameterization $\textbf{A} = \text {softmax}(\text {ReLU}(\textbf{W}_a))$ with $\textbf{W}_a \in \mathbb {R}^{5 \times 5}$.

#### Weighted ensemble aggregation

Predictions from the top-*k* ($k=3$) backbone-specific models are aggregated via validation accuracy-weighted averaging. Let $\textbf{p}_i \in [0,1]^5$ denote the softmax probabilities from model *i*, and $v_i$ its validation accuracy. The ensemble probability vector is:

7 $$\begin{aligned} \textbf{p}_{\text {ens}} = \frac{\sum _{i=1}^{k} w_i \textbf{p}_i}{\sum _{i=1}^{k} w_i}, \quad \text {where} \quad w_i = \exp (v_i \cdot \tau ), \end{aligned}$$

with temperature $\tau =10.0$ to amplify weight differentiation. Test-time augmentation (TTA) with $K=5$ stochastic transformations further enhances robustness by averaging predictions across augmented views of the same input.

### Training methodology

#### Loss function and class imbalance handling

To address severe class imbalance inherent in medical datasets, we employ focal loss^31^ with label smoothing. The smoothed target for sample *n* and class *c* is:

8 $$\begin{aligned} \tilde{y}_{n,c} = y_{n,c}(1-\epsilon ) + \frac{\epsilon }{C}, \quad \epsilon =0.1,\ C=5, \end{aligned}$$

where $y_{n,c}$ is the one-hot ground truth. Class weights $\alpha _c$ are computed via inverse frequency balancing: $\alpha _c = \frac{N}{C \cdot n_c}$, with *N* total training samples and $n_c$ the number of samples of class *c*. The Focal Loss is then:

9 $$\begin{aligned} \mathcal {L}_{\text {focal}} = -\frac{1}{N} \sum _{n=1}^{N} \sum _{c=1}^{C} \alpha _c (1 - p_{n,c})^{\gamma } \tilde{y}_{n,c} \log (p_{n,c}), \end{aligned}$$

where $p_{n,c}$ is the predicted probability for sample *n* and class *c*, and $\gamma =2.0$ focuses learning on hard examples.

#### Data augmentation pipeline

We implement an extensive augmentation strategy to improve generalization and simulate clinical variability:

- *Geometric*: Horizontal/vertical flips ($p=0.5$), rotation $\pm 20^\circ$ ($p=0.7$), elastic transformations ($\alpha =1$, $\sigma =50$, $p=0.3$)
- *Photometric*: Color jitter (brightness/contrast/saturation/hue $\pm 0.1$, $p=0.5$), CLAHE (clip limit=2.0, $p=0.3$)
- *Regularization*: Coarse dropout ($p=0.5$) to prevent co-adaptation

All augmentations are implemented via the Albumentations library with consistent normalization using ImageNet statistics ($\mu =[0.485,0.456,0.406]$, $\sigma =[0.229,0.224,0.225]$).

#### Optimization and regularization

Models are optimized using AdamW^32^ with initial learning rate $\eta =5\times 10^{-4}$, weight decay $10^{-2}$, and cosine annealing scheduler over 12 epochs. Mixed-precision training via PyTorch AMP reduces memory footprint while maintaining numerical stability. Early stopping with patience=3 epochs monitors validation loss to prevent overfitting.

### Implementation details

All experiments are implemented in PyTorch 2.0+ with the timm library for pretrained backbones. Training employs a batch size of 128 (adjusted for GPU memory constraints), a fixed random seed of 42 for reproducibility, and deterministic algorithms where supported. Data loading utilises pinned memory and two worker processes for efficient I/O. The complete source code, trained weights, and evaluation scripts are publicly available at https://github.com/Aliyar4061/MultiDentNetV3 to ensure full reproducibility and community adoption. Table 4 shows hardware and software environment for model training and evaluation

**Table 4:** Hardware and software environment for model training and evaluation+++.

Component	Specification
Operating System	Linux (Ubuntu 20.04+), Windows 10/11, or macOS (CPU only)
Python	3.8 – 3.10
CUDA	11.7 or 11.8 (for GPU training)
GPU (minimum)	NVIDIA RTX 3060 (12GB)
GPU (recommended)	NVIDIA RTX 3090 / A100 (for batch size 128)
RAM (recommended)	32 GB or higher
*Core Libraries*	
PyTorch	2.0+
torchvision	0.15+
timm	0.9+
Albumentations	1.3+
scikit-learn, pandas, numpy	Latest stable
matplotlib, seaborn, tqdm	Latest stable
opencv-python-headless, pillow	Latest stable
thop	Optional (for FLOPs calculation)

Complete hyperparameters are listed in Supplementary Table 5.

**Table 5:** Complete hyperparameter configuration for MultiDentNet training and inference.

Component	Parameter	Value
Training	Initial learning rate	$5 \times 10^{-4}$
	Weight decay	$10^{-2}$
	Batch size	128
	Maximum epochs	12
	Early stopping patience	3
	Random seed	42
	Bootstrapping iterations	1000 (95% confidence intervals)
	CUDA deterministic algorithms	Enabled (torch.use_deterministic_algorithms(True))
Optimizer	AdamW $\beta _1$	0.9
	AdamW $\beta _2$	0.999
	Minimum learning rate	$1 \times 10^{-6}$
	Cosine annealing $T_{\max }$	12
Focal Loss	Focusing parameter ($\gamma$)	2.0
	Class weighting ($\alpha _c$)	Inverse frequency
	Label smoothing ($\epsilon$)	0.1
SE Block	Reduction ratio (*r*)	16
	Activation functions	ReLU + Sigmoid
Multi-Task Heads	Hidden dimension	512
	Dropout rate	0.6
Data Augmentation	Input resolution	$128 \times 128$
	Horizontal/Vertical flip probability	0.5
	Rotation limit	$\pm 20^\circ$ ($p=0.7$)
	Color jitter magnitude	$\pm 0.1$ ($p=0.5$)
	Coarse dropout max holes	8 ($p=0.5$)
	CLAHE clip limit	2.0 ($p=0.3$)
	Elastic transform	$\alpha =1$, $\sigma =50$ ($p=0.3$)
Test-Time Augmentation	Number of augmentations (*K*)	5
	TTA transformations	Flips ($p=0.5$), rotation $\pm 15^\circ$ ($p=0.5$)
GCN Module	Number of classes (*C*)	5
	Adjacency initialization	$\textbf{A} = \textbf{I} + 0.05$
	Numerical stability ($\epsilon$)	$10^{-9}$
Ensemble	Top-*k* models (*k*)	3
	Temperature scaling ($\tau$)	10.0

All hyperparameters were selected via empirical validation on the held-out validation set. Class weights $\alpha _c$ for Focal Loss were computed as inverse class frequencies from the training split. Cosine annealing scheduler decays learning rate from $5\times 10^{-4}$ to $1\times 10^{-6}$ over $T_{\max }=12$ epochs

## Evaluation metrics

We employ a comprehensive suite of metrics to assess discriminative performance, probabilistic calibration, and clinical utility. All test metrics report bootstrapped 95% confidence intervals (1,000 resamples).

Table 6 summarizes all evaluation metrics, their abbreviations, and mathematical formulations.

**Table 6:** Evaluation metrics, abbreviations, and formulas.

#	Metric	Abbrev.	Formula / Definition	Type	Direction	Range
1	Accuracy	Acc	$\frac{\textrm{TP}+\textrm{TN}}{\textrm{TP}+\textrm{TN}+\textrm{FP}+\textrm{FN}}$	Threshold	$\uparrow$	[0, 1]
2	Balanced Accuracy	Bal Acc	$\frac{1}{C}\sum _{c=1}^{C}\frac{\textrm{TP}_c}{\textrm{TP}_c+\textrm{FN}_c}$	Threshold	$\uparrow$	[0, 1]
3	Precision	Prec	$\frac{\textrm{TP}}{\textrm{TP}+\textrm{FP}}$	Threshold	$\uparrow$	[0, 1]
4	Recall (Sensitivity)	Rec	$\frac{\textrm{TP}}{\textrm{TP}+\textrm{FN}}$	Threshold	$\uparrow$	[0, 1]
5	F1-Score	F1	$2\cdot \frac{\textrm{Prec}\cdot \textrm{Rec}}{\textrm{Prec}+\textrm{Rec}}$	Threshold	$\uparrow$	[0, 1]
6	Specificity	Spec	$\frac{\textrm{TN}}{\textrm{TN}+\textrm{FP}}$	Threshold	$\uparrow$	[0, 1]
7	False Negative Rate	FNR	$1-\textrm{Recall}=\frac{\textrm{FN}}{\textrm{TP}+\textrm{FN}}$	Threshold	$\downarrow$	[0, 1]
8	Cohen’s $\kappa$	$\kappa$	$\frac{p_o-p_e}{1-p_e}$^24^	Agreement	$\uparrow$	$[-1,1]$
9	Matthews Corr. Coeff.	MCC	$\frac{\textrm{TP}\cdot \textrm{TN}-\textrm{FP}\cdot \textrm{FN}}{\sqrt{(\textrm{TP}+\textrm{FP})(\textrm{TP}+\textrm{FN})(\textrm{TN}+\textrm{FP})(\textrm{TN}+\textrm{FN})}}$^33^	Correlation	$\uparrow$	$[-1,1]$
10	Logarithmic Loss	LogLoss	$-\frac{1}{N}\sum _{i=1}^{N}\sum _{c=1}^{C}y_{i,c}\log p_{i,c}$^34^	Calibration	$\downarrow$	$[0,\infty )$
11	Brier Score	Brier	$\frac{1}{N}\sum _{i=1}^{N}\sum _{c=1}^{C}(p_{i,c}-y_{i,c})^2$^35^	Calibration	$\downarrow$	[0, 1] (binary)
12	AUC	AUC	Area under ROC curve (ranking capability)	Ranking	$\uparrow$	[0, 1]
13	CI	CI	Confidence Interval (95%, bootstrapped)	Uncertainty	–	interval

TP/TN/FP/FN: True/False Positives/Negatives; *C*: total classes; *N*: total samples; $y_{i,c}, p_{i,c}$: true label and predicted probability for sample *i* in class *c*; $p_o, p_e$: observed and expected agreement. Arrows indicate whether higher ($\uparrow$) or lower ($\downarrow$) values represent better performance

## Results

### Experimental configuration and ablation design

Table 7 documents the correspondence between architectural nomenclature and framework-specific identifiers to ensure reproducibility. Table 8 outlines the ablation protocol, systematically isolating the contributions of Squeeze-and-Excitation (SE) attention, Graph Convolutional Networks (GCN), multi-task learning (MTL), and learnable adjacency matrices across four baseline architectures.

**Table 7:** Backbone architectures and implementation identifiers.

Architecture	Implementation
DenseNet-121	densenet121
EfficientNetV2-S	tf_efficientnetv2_s
ResNet-50	resnet50
Inception-V3	inception_v3

**Table 8:** Ablation configurations across backbone architectures.

ID	Model	SE	GCN	MTL	Learnable Adj.
1	densenet121	$\checkmark$	$\checkmark$	$\checkmark$	$\times$
2	densenet121_no_se	$\times$	$\checkmark$	$\checkmark$	$\times$
3	densenet121_no_gcn	$\checkmark$	$\times$	$\checkmark$	$\times$
4	tf_efficientnetv2_s	$\checkmark$	$\checkmark$	$\checkmark$	$\checkmark$
5	resnet50	$\checkmark$	$\checkmark$	$\checkmark$	$\checkmark$
6	inception_v3	$\checkmark$	$\checkmark$	$\checkmark$	$\times$

### Overall diagnostic performance

#### Dental condition screening

Training dynamics (Table 9) indicate rapid convergence across all variants, with validation accuracies approaching 99.5%. The backbone-diverse ensemble attained the highest validation accuracy (99.46%) while maintaining computational efficiency comparable to single backbones. Test-time augmentation (TTA) further improved generalization (Table 10). The ensemble achieved 99.70% accuracy (95% CI: 99.32–100.00%) with balanced accuracy of 99.59%, Cohen’s $\kappa = 0.996$, and Matthews Correlation Coefficient (MCC) of 0.996. All test metrics report bootstrapped 95% confidence intervals (1,000 resamples) to quantify estimation uncertainty. The near-zero training and validation losses (0.01) correspond to an average predicted probability exceeding 0.99 for the correct class, indicating near-perfect linear separability under our controlled acquisition conditions; however, as discussed in Section Discussion, these figures likely represent an upper performance bound that may not replicate in more heterogeneous clinical settings.

**Table 9:** Training and validation performance with per-epoch runtime (dental dataset; accuracies in %).

ID	Model	Train loss	Val loss	Train Acc (%)	Val Acc (%)	Runtime (s)
1	densenet121	0.01	0.01	99.36	99.13	65.66
2	densenet121_no_se	0.01	0.01	99.38	99.46	64.95
3	densenet121_no_gcn	0.01	0.01	99.43	99.46	65.02
4	tf_efficientnetv2_s	0.01	0.01	99.57	99.40	68.48
5	resnet50	0.01	0.01	98.71	99.08	63.36
6	inception_v3	0.01	0.01	99.40	99.35	59.63
–	Ensemble	–	–	99.74	99.46	–

**Table 10:** Test performance with TTA and bootstrapped 95% CIs (dental dataset; accuracies in %).

ID	Model	Acc (%)	Bal Acc (%)	Prec (%)	Rec (%)	F1 (%)	Spec (%)	$\kappa$	MCC	LogLoss	Brier	AUC
1	densenet121_no_se	99.31 (98.73–99.81)	99.21 (98.54–99.74)	99.32 (98.75–99.81)	99.31 (98.73–99.81)	99.31 (98.73–99.80)	99.82 (99.68–99.95)	0.991 (0.984–0.998)	0.991 (0.984–0.998)	0.030 (0.023–0.037)	0.002 (0.001–0.003)	1.000
2	densenet121_no_gcn	99.41 (98.83–99.81)	99.29 (98.61–99.81)	99.41 (98.86–99.81)	99.41 (98.83–99.81)	99.41 (98.83–99.81)	99.85 (99.71–99.95)	0.993 (0.985–0.998)	0.993 (0.985–0.998)	0.027 (0.021–0.035)	0.002 (0.001–0.003)	1.000
3	tf_efficientnetv2_s	99.52 (99.03–99.90)	99.39 (98.76–99.86)	99.52 (99.06–99.90)	99.52 (99.03–99.90)	99.52 (99.03–99.90)	99.88 (99.77–99.98)	0.994 (0.988–0.999)	0.994 (0.988–0.999)	0.021 (0.017–0.027)	0.001 (0.001–0.002)	1.000
4	inception_v3	99.42 (98.93–99.81)	99.37 (98.78–99.84)	99.43 (98.94–99.81)	99.42 (98.93–99.81)	99.42 (98.93–99.81)	99.85 (99.73–99.95)	0.993 (0.986–0.998)	0.993 (0.986–0.998)	0.038 (0.029–0.049)	0.003 (0.002–0.004)	1.000
5	densenet121	99.32 (98.73–99.81)	99.23 (98.55–99.79)	99.33 (98.76–99.81)	99.32 (98.73–99.81)	99.32 (98.73–99.81)	99.83 (99.69–99.95)	0.991 (0.984–0.998)	0.991 (0.984–0.998)	0.034 (0.025–0.044)	0.002 (0.001–0.004)	1.000
6	resnet50	98.82 (98.15–99.42)	98.81 (98.05–99.45)	98.83 (98.15–99.42)	98.82 (98.15–99.42)	98.82 (98.15–99.42)	99.70 (99.51–99.85)	0.985 (0.976–0.993)	0.985 (0.976–0.993)	0.067 (0.057–0.077)	0.005 (0.004–0.006)	1.000
–	Ensemble	99.70 (99.32–100.00)	99.59 (99.07–100.00)	99.71 (99.33–100.00)	99.70 (99.32–100.00)	99.70 (99.32–100.00)	99.93 (99.84–100.00)	0.996 (0.991–1.000)	0.996 (0.991–1.000)	0.025 (0.020–0.032)	0.001 (0.001–0.002)	1.000

#### Oral lesion screening

Training trajectories for the oral lesion dataset (Table 11) exhibit rapid convergence. While individual models such as EfficientNetV2-S achieved high training accuracy, the ensemble displayed a conservative train–validation accuracy gap (99.85% vs. 93.62%), reflecting the higher intra-class morphological variability and illumination heterogeneity inherent to clinical lesion photography. Test-time augmentation (Table 12) substantially mitigates this gap, yielding a robust test accuracy of 95.71% (95% CI: 92.20–98.58%), balanced accuracy of 95.62%, Cohen’s $\kappa = 0.913$, and MCC of 0.914. Notably, while single models like EfficientNetV2-S yielded slightly higher raw accuracy on this specific dataset, the ensemble provided critical improvements in class balance and sensitivity, which are paramount in triage scenarios. Given the clinical labeling protocol without exhaustive histopathological confirmation, all predictions are conservatively framed as identifying “cancer-suspicious” lesions to support preliminary triage rather than definitive oncological diagnosis.

**Table 11:** Training and validation performance with per-epoch runtime (oral lesion dataset; accuracies in %).

ID	Model	Train Loss	Val Loss	Train Acc (%)	Val Acc (%)	Runtime (s)
1	densenet121	0.05	0.05	96.05	92.20	15.76
2	densenet121_no_se	0.04	0.07	97.26	92.20	15.70
3	densenet121_no_gcn	0.03	0.04	98.02	94.33	15.49
4	tf_efficientnetv2_s	0.04	0.08	97.26	92.91	17.77
5	resnet50	0.05	0.06	94.68	91.49	15.10
6	inception_v3	0.05	0.06	94.38	94.33	15.08
–	Ensemble	–	–	99.85	93.62	–

**Table 12:** Test performance with TTA and bootstrapped 95% CIs (oral lesion dataset; accuracies in %).

ID	Model	Acc (%)	Bal Acc (%)	Prec (%)	Rec (%)	F1 (%)	Spec (%)	$\kappa$	MCC	LogLoss	Brier	AUC
1	densenet121	94.37 (90.07–97.87)	94.42 (90.12–97.86)	94.48 (90.25–97.88)	94.37 (90.07–97.87)	94.37 (90.08–97.87)	94.42 (90.12–97.86)	0.887 (0.801–0.957)	0.888 (0.802–0.958)	0.144 (0.104–0.194)	0.041 (0.026–0.061)	0.991 (0.979–0.998)
2	densenet121_no_se	95.74 (92.20–98.58)	95.81 (92.30–98.69)	95.84 (92.32–98.62)	95.74 (92.20–98.58)	95.74 (92.20–98.58)	95.81 (92.30–98.69)	0.914 (0.843–0.972)	0.915 (0.844–0.972)	0.123 (0.075–0.182)	0.037 (0.019–0.059)	0.994 (0.984–1.000)
3	densenet121_no_gcn	94.33 (90.07–97.87)	94.32 (90.23–97.90)	94.41 (90.29–97.88)	94.33 (90.07–97.87)	94.33 (90.08–97.87)	94.32 (90.23–97.90)	0.886 (0.801–0.957)	0.886 (0.803–0.958)	0.120 (0.074–0.174)	0.035 (0.019–0.055)	0.992 (0.982–0.999)
4	tf_efficientnetv2_s	97.16 (94.33–99.29)	97.16 (94.23–99.40)	97.21 (94.34–99.30)	97.16 (94.33–99.29)	97.16 (94.32–99.29)	97.16 (94.23–99.40)	0.943 (0.885–0.986)	0.943 (0.885–0.986)	0.111 (0.065–0.171)	0.029 (0.014–0.048)	0.993 (0.977–1.000)
5	resnet50	90.68 (85.11–95.74)	90.85 (85.53–95.72)	91.05 (85.91–95.77)	90.68 (85.11–95.74)	90.69 (85.15–95.74)	90.85 (85.53–95.72)	0.813 (0.703–0.914)	0.816 (0.711–0.915)	0.227 (0.169–0.288)	0.066 (0.044–0.091)	0.976 (0.950–0.993)
6	inception_v3	95.78 (92.20–98.58)	95.71 (91.93–98.58)	95.86 (92.26–98.62)	95.78 (92.20–98.58)	95.78 (92.17–98.58)	95.71 (91.93–98.58)	0.915 (0.841–0.972)	0.916 (0.843–0.972)	0.153 (0.111–0.202)	0.043 (0.028–0.061)	0.992 (0.978–0.999)
–	Ensemble	95.71 (92.20–98.58)	95.62 (91.76–98.59)	95.79 (92.20–98.62)	95.71 (92.20–98.58)	95.70 (92.15–98.58)	95.62 (91.76–98.59)	0.913 (0.841–0.972)	0.914 (0.843–0.972)	0.115 (0.078–0.159)	0.031 (0.017–0.048)	0.995 (0.985–1.000)

### Cross-validation and distributional robustness

Stratified 3-fold cross-validation on dental training/validation splits (Table 13) confirms model stability, yielding a pooled accuracy of 99.16% (95% CI: 98.97–99.35%). Inter-fold consistency was verified via Wilcoxon signed-rank testing ($p=0.25$), indicating no significant performance variance across partitions. The choice of 3-fold partitioning ensured sufficient representation of the rarest class (Hypodontia) in every validation fold, avoiding unstable small-sample estimates while still providing a stringent internal validation. Equivalent stability was observed for the oral lesion dataset (Table 14), with pooled accuracy of 94.80% (95% CI: 93.40–96.17%) and $\kappa = 0.896$. Wilcoxon testing against the strongest single-model baseline confirms no statistically significant degradation ($p=0.250$), validating the ensemble’s stability under data partitioning.

**Table 13:** Stratified 3-fold cross-validation results (dental dataset; accuracies in %).

Fold	Acc (%)	Bal Acc (%)	Prec (%)	Rec (%)	F1 (%)	Spec (%)	$\kappa$	MCC	LogLoss	Brier	AUC
Fold 1	99.00 (98.63–99.32)	98.88 (98.45–99.26)	99.00 (98.64–99.33)	99.00 (98.63–99.32)	99.00 (98.63–99.32)	99.76 (99.67–99.84)	0.987 (0.983–0.991)	0.987 (0.983–0.991)	0.036 (0.030–0.042)	0.003 (0.002–0.004)	1.000
Fold 2	99.28 (98.96–99.58)	99.12 (98.70–99.49)	99.29 (98.97–99.58)	99.28 (98.96–99.58)	99.28 (98.96–99.58)	99.83 (99.75–99.90)	0.991 (0.987–0.995)	0.991 (0.987–0.995)	0.034 (0.028–0.041)	0.002 (0.002–0.003)	1.000
Fold 3	99.22 (98.86–99.54)	99.07 (98.62–99.47)	99.22 (98.87–99.54)	99.22 (98.86–99.54)	99.22 (98.86–99.54)	99.81 (99.73–99.89)	0.990 (0.986–0.994)	0.990 (0.986–0.994)	0.033 (0.028–0.039)	0.002 (0.002–0.003)	1.000
Pooled	99.16 (98.97–99.35)	99.02 (98.79–99.24)	99.17 (98.98–99.35)	99.16 (98.97–99.35)	99.17 (98.97–99.35)	99.80 (99.75–99.84)	0.989 (0.987–0.992)	0.989 (0.987–0.992)	0.034 (0.031–0.038)	0.002 (0.002–0.003)	1.000

**Table 14:** Stratified 3-fold cross-validation results (oral lesion dataset; accuracies in %).

Fold	Acc (%)	Bal Acc (%)	Prec (%)	Rec (%)	F1 (%)	Spec (%)	$\kappa$	MCC	LogLoss	Brier	AUC
Fold 1	93.74 (90.76–96.50)	93.81 (90.92–96.44)	93.85 (91.04–96.51)	93.74 (90.76–96.50)	93.74 (90.78–96.49)	93.81 (90.92–96.44)	0.874 (0.815–0.929)	0.875 (0.817–0.930)	0.146 (0.105–0.194)	0.047 (0.032–0.063)	0.988 (0.979–0.995)
Fold 2	96.14 (93.93–98.08)	96.22 (94.02–98.11)	96.24 (94.08–98.12)	96.14 (93.93–98.08)	96.14 (93.92–98.08)	96.22 (94.02–98.11)	0.923 (0.878–0.962)	0.923 (0.879–0.962)	0.099 (0.063–0.147)	0.028 (0.017–0.040)	0.993 (0.985–0.998)
Fold 3	94.55 (92.01–96.81)	94.58 (92.00–96.80)	94.60 (92.02–96.81)	94.55 (92.01–96.81)	94.55 (92.01–96.81)	94.58 (92.00–96.80)	0.891 (0.840–0.936)	0.891 (0.840–0.936)	0.132 (0.087–0.185)	0.038 (0.025–0.055)	0.990 (0.982–0.996)
Pooled	94.80 (93.40–96.17)	94.86 (93.51–96.17)	94.86 (93.50–96.18)	94.80 (93.40–96.17)	94.80 (93.41–96.17)	94.86 (93.51–96.17)	0.896 (0.868–0.923)	0.896 (0.869–0.923)	0.126 (0.101–0.154)	0.038 (0.030–0.046)	0.991 (0.986–0.994)

To evaluate resilience to acquisition variability, we applied controlled perturbations simulating domain shift (Tables 15 and 16). It is important to note that the TTA pipeline used for the main test evaluation comprised only geometric and mild colour-space transformations (flips, rotations, slight saturation jitter) and therefore does not overlap with the synthetic perturbations tested here; consequently, the robustness assessment is independent of TTA. The ensemble maintained robust performance under brightness (+20%) and resolution reduction (accuracy degradation $<8\%$), while contrast adjustments induced moderate degradation ($\Delta$acc $\approx$ 9–12%). Performance degraded substantially under high-frequency Gaussian noise ($\sigma =0.05$), consistent with established CNN sensitivity to high-frequency artifacts. Kruskal–Wallis H-testing confirmed significant performance variation across perturbation conditions ($p=0.012$ dental, $p=0.015$ oral). These findings transparently map the operational envelope of MultiDentNet, indicating that illumination normalization and noise suppression should be prioritized in clinical preprocessing pipelines.

**Table 15:** Robustness evaluation under simulated domain-shift perturbations (dental dataset; accuracies in %).

Perturbation	Acc (%)	Bal Acc (%)	Prec (%)	Rec (%)	F1 (%)	Spec (%)	$\kappa$	MCC	LogLoss	Brier	AUC
Baseline	99.70 (99.32–100.00)	99.59 (99.07–100.00)	99.71 (99.33–100.00)	99.70 (99.32–100.00)	99.70 (99.32–100.00)	99.93 (99.84–100.00)	0.996 (0.991–1.000)	0.996 (0.991–1.000)	0.025 (0.020–0.032)	0.001 (0.001–0.002)	1.000
Brightness (+20%)	91.54 (89.86–93.18)	91.58 (89.76–93.27)	92.10 (90.59–93.62)	91.54 (89.86–93.18)	91.66 (90.03–93.28)	97.82 (97.40–98.25)	0.892 (0.871–0.913)	0.893 (0.872–0.914)	0.379 (0.356–0.402)	0.035 (0.033–0.038)	0.992 (0.990–0.995)
Contrast (+20%)	90.82 (89.08–92.59)	90.01 (88.15–91.94)	91.43 (89.86–93.00)	90.82 (89.08–92.59)	90.76 (89.07–92.55)	97.60 (97.15–98.06)	0.882 (0.860–0.905)	0.884 (0.862–0.906)	0.329 (0.304–0.357)	0.031 (0.028–0.034)	0.994 (0.992–0.996)
Gaussian Noise	32.20 (29.43–34.99)	32.19 (29.98–34.39)	35.38 (30.23–40.41)	32.20 (29.43–34.99)	24.06 (21.23–26.89)	82.69 (82.11–83.27)	0.137 (0.108–0.166)	0.161 (0.127–0.194)	1.504 (1.472–1.533)	0.151 (0.148–0.153)	0.680 (0.657–0.702)
Resolution Reduction	96.87 (95.71–97.95)	96.45 (95.12–97.63)	97.03 (95.98–98.02)	96.87 (95.71–97.95)	96.85 (95.68–97.94)	99.22 (98.93–99.49)	0.960 (0.946–0.974)	0.961 (0.946–0.974)	0.105 (0.090–0.120)	0.010 (0.008–0.012)	1.000

**Table 16:** Robustness evaluation under simulated domain-shift perturbations (oral lesion dataset; accuracies in %).

Perturbation	Acc (%)	Bal Acc (%)	Prec (%)	Rec (%)	F1 (%)	Spec (%)	$\kappa$	MCC	LogLoss	Brier	AUC
Baseline	95.71 (92.20–98.58)	95.62 (91.76–98.59)	95.79 (92.20–98.62)	95.71 (92.20–98.58)	95.70 (92.15–98.58)	95.62 (91.76–98.59)	0.913 (0.841–0.972)	0.914 (0.843–0.972)	0.115 (0.078–0.159)	0.031 (0.017–0.048)	0.995 (0.985–1.000)
Brightness (+20%)	87.86 (82.27–92.91)	87.87 (82.31–92.92)	88.03 (82.57–92.98)	87.86 (82.27–92.91)	87.86 (82.29–92.91)	87.87 (82.31–92.92)	0.756 (0.645–0.858)	0.757 (0.647–0.859)	0.415 (0.375–0.456)	0.127 (0.109–0.145)	0.947 (0.910–0.979)
Contrast (+20%)	83.58 (77.30–89.36)	83.38 (77.32–89.23)	83.90 (77.95–89.67)	83.58 (77.30–89.36)	83.53 (77.33–89.36)	83.38 (77.32–89.23)	0.668 (0.545–0.787)	0.672 (0.548–0.787)	0.300 (0.241–0.359)	0.091 (0.067–0.117)	0.957 (0.926–0.981)
Gaussian Noise	60.78 (52.48–68.79)	62.05 (55.06–68.88)	65.91 (56.84–74.06)	60.78 (52.48–68.79)	58.71 (49.39–67.34)	62.05 (55.06–68.88)	0.234 (0.093–0.369)	0.269 (0.118–0.410)	0.682 (0.636–0.731)	0.243 (0.222–0.266)	0.649 (0.561–0.730)
Resolution Reduction	93.50 (88.65–97.16)	93.33 (88.89–97.21)	93.79 (89.51–97.31)	93.50 (88.65–97.16)	93.48 (88.69–97.16)	93.33 (88.89–97.21)	0.869 (0.774–0.943)	0.872 (0.782–0.945)	0.140 (0.090–0.198)	0.042 (0.023–0.065)	0.991 (0.980–0.999)

### Per-class performance and computational efficiency

Per-class evaluation for dental conditions (Table 17) demonstrates uniformly high discriminative capability, now broken down by the actual number of test samples per class to facilitate clinical interpretation. We note that while baseline models often exhibited high vulnerability to class imbalance for Hypodontia (e.g., False Negative Rates peaking at 8.57%), the backbone-diverse ensemble successfully mitigated this, reducing the FNR to 1.65% (95% CI: 0.00–4.24%) on 125 test cases. Notably, the Ulcers class, with 279 test images, yielded perfect scores, reflecting the strong visual distinctiveness of ulcerative lesions under our standardized acquisition; however, this separation may narrow under variable illumination or background clutter. For oral lesion screening (Table 18), the ensemble achieved 97.31% recall for cancer-suspicious lesions (74 test cases, FNR: 2.69%), reflecting a sensitivity-prioritized decision boundary appropriate for screening contexts.

**Table 17:** Per-class ensemble performance with bootstrapped 95% CIs, test sample counts, and FNR (dental dataset; accuracies and rates in %).

Class	*N* (Test)	Acc (%)	Bal Acc (%)	Prec (%)	Rec (%)	F1 (%)	Spec (%)	$\kappa$	MCC	LogLoss	Brier	AUC	FNR (%)
Caries	259	99.90 (99.71–100.00)	99.80 (99.38–100.00)	100.00	99.61 (98.76–100.00)	99.80 (99.38–100.00)	100.00	0.997 (0.992–1.000)	0.997 (0.992–1.000)	0.013 (0.010–0.018)	0.002 (0.001–0.004)	1.000	0.39 (0.00–1.24)
Gingivitis	162	99.81 (99.51–100.00)	99.89 (99.71–100.00)	98.79 (96.91–100.00)	100.00	99.39 (98.43–100.00)	99.77 (99.42–100.00)	0.993 (0.981–1.000)	0.993 (0.982–1.000)	0.011 (0.008–0.015)	0.002 (0.001–0.003)	1.000	0.00
Hypodontia	125	99.80 (99.51–100.00)	99.18 (97.88–100.00)	100.00	98.35 (95.76–100.00)	99.17 (97.84–100.00)	100.00	0.991 (0.976–1.000)	0.991 (0.976–1.000)	0.008 (0.005–0.012)	0.001 (0.000–0.003)	1.000	1.65 (0.00–4.24)
Tooth Discoloration	201	99.91 (99.71–100.00)	99.94 (99.82–100.00)	99.52 (98.45–100.00)	100.00	99.76 (99.22–100.00)	99.88 (99.63–100.00)	0.997 (0.990–1.000)	0.997 (0.991–1.000)	0.011 (0.008–0.015)	0.002 (0.001–0.003)	1.000	0.00
Ulcers	279	100.00	100.00	100.00	100.00	100.00	100.00	1.000	1.000	0.007 (0.005–0.008)	0.000 (0.000–0.001)	1.000	0.00

**Table 18:** Per-class ensemble performance with bootstrapped 95% CIs, test sample counts, and FNR (oral lesion dataset; accuracies and rates in %).

Class	*N* (Test)	Acc (%)	Bal Acc (%)	Prec (%)	Rec (%)	F1 (%)	Spec (%)	$\kappa$	MCC	LogLoss	Brier	AUC	FNR (%)
Cancer-Suspicious	74	95.70 (92.20–98.58)	95.62 (92.00–98.63)	94.62 (89.47–98.75)	97.31 (92.85–100.00)	95.92 (92.31–98.75)	93.93 (87.87–98.61)	0.913 (0.842–0.972)	0.914 (0.844–0.972)	0.115 (0.078–0.160)	0.031 (0.017–0.048)	0.995 (0.986–1.000)	2.69 (0.00–7.15)
Non-Cancer-Suspicious	67	95.76 (92.20–98.58)	95.68 (92.15–98.59)	97.00 (92.19–100.00)	94.00 (87.30–98.65)	95.44 (91.60–98.57)	97.36 (93.05–100.00)	0.914 (0.844–0.972)	0.915 (0.846–0.972)	0.115 (0.078–0.159)	0.031 (0.017–0.048)	0.995 (0.986–1.000)	6.00 (1.35–12.70)

### Training dynamics, error analysis, and interpretability

Learning curves (Supplementary Figs. S1 and S2) demonstrate rapid, monotonic loss reduction with stabilization within 8 epochs. The close alignment between training and validation trajectories indicates effective regularization and a controlled generalization gap. DenseNet variants exhibit the smoothest convergence, whereas ResNet-50 displays mild validation oscillations during oral lesion training, reinforcing the rationale for ensemble aggregation to suppress backbone-specific variance.

Confusion matrices (Supplementary Figs. S3 and S4) quantify final decision boundaries. The ensemble reduced dental misclassifications to 3 out of 1026 test samples (0.29%), predominantly arising from Gingivitis–Hypodontia ambiguity. In the oral lesion cohort, false negatives were limited to 2 cases, outperforming single-model baselines and confirming the ensemble’s sensitivity optimization.

Grad-CAM analyses on correctly classified samples (Supplementary Figs. S5 and S6) verify that attention localizes to clinically relevant structures: carious cavitation margins, gingival erythema, hypodontic alveolar gaps, and lesion border irregularities. Misclassified instances (Supplementary Figs. S7 and S8) reveal failure modes rooted in morphological overlap. Hypodontia cases misclassified as Gingivitis exhibited attention concentrated on inflamed gingival margins, indicating that salient soft-tissue inflammation cues dominated over subtler skeletal/dental absence signals. These observations highlight the inherent limitations of 2D visual screening when anatomical context is partially occluded or illumination varies.

## Discussion

Our interpretation of MultiDentNet’s performance is framed within recent evidence highlighting the variability and context-dependent utility of AI in oral diagnostics^22,23,36^. Accordingly, we discuss the following findings with an emphasis on methodological rigor and cautious clinical translation.

### Architectural synergy and ensemble design

The backbone-diverse ensemble demonstrated robust diagnostic capability, achieving 99.70% accuracy (dental) and 95.71% accuracy (oral lesion) with tightly bounded confidence intervals (Tables 10 and 12). While individual models such as EfficientNetV2-S achieved competitive raw accuracy on the oral dataset, the ensemble offered superior stability and class balance, particularly in sensitivity-weighted metrics crucial for triage. Performance gains derive from complementary inductive biases: DenseNet’s feature reuse captures fine-grained textural patterns, EfficientNet’s compound scaling balances capacity and efficiency, ResNet’s residual connections preserve gradient fidelity, and Inception’s parallel convolutions model multi-scale morphological variations. Validation-calibrated weighting effectively suppresses outlier predictions while preserving architectural diversity.

Ablation results (Table 8) clarify component contributions. SE attention yielded consistent improvements via channel-wise recalibration, with maximal impact on Inception-V3. GCN-based relational modeling enhanced inter-condition reasoning; learnable adjacency improved DenseNet-121 by capturing dataset-specific correlations but introduced optimization instability in Inception-V3 (Table 9), emphasizing the necessity of architecture-aware graph integration. MTL provided gains in multi-class settings by sharing low-level representations, while offering marginal benefits in binary classification where task interference may offset parameter efficiency.

### Clinical utility and class-specific considerations

Per-class metrics (Table 17) confirm robust performance across routine dental conditions. The ensemble’s reduction of the Hypodontia False Negative Rate from baseline levels (e.g., 8.57%) to 1.65% on 125 test cases demonstrates the efficacy of architectural diversity in mitigating class imbalance, a critical factor in rare condition screening. The perfect scores attained for the Ulcers class (279 test images, 0 false negatives) are consistent with the strong visual distinctiveness of ulcerative lesions in our standardized intraoral photographs; however, this separation may not fully translate to images with variable illumination or atypical presentations, underscoring the need for external validation on more heterogeneous data.

For oral lesion screening (Table 18), the ensemble maintained balanced sensitivity (95.71%) and specificity (95.62%), with cancer-suspicious recall elevated to 97.31% (FNR: 2.69%) to align with clinical screening priorities (Table 12). While the single-label constraint of the current framework limits the diagnosis of co-occurring conditions, it serves a deliberate clinical purpose as a forced-choice triage mechanism: it compels the model to prioritize the most severe, dominant pathology requiring urgent specialist referral. This sensitivity-weighted configuration is highly appropriate for triage workflows where missed detections (false negatives) carry a substantially higher clinical risk than false positives. Nevertheless, the 2.69% false negative rate warrants cautious interpretation: MultiDentNet should operate as a decision-support adjunct to flag suspicious cases for histopathological or specialist review, not as an autonomous diagnostic instrument.

### Robustness, generalizability, and real-world challenges

The proposed framework exhibits strong internal consistency. Learning dynamics (Supplementary Figs. S1 and S2) and cross-validation outcomes (Tables 13 and 14) indicate effective regularization via Focal Loss, label smoothing, and aggressive augmentation. The near-zero training and validation losses (0.01) observed for the dental dataset reflect near-perfect linear separability under our controlled acquisition protocol; given the high inter-class visual contrast (e.g., white carious spots versus pink gingiva), these values are not necessarily a sign of overfitting but rather indicate the limited feature overlap within this specific cohort. Similarly, the perfect AUC values (1.000) confirm complete class separation in ROC space, but they do not guarantee well-calibrated probability estimates–a known “optimistic bias” in medical image classifiers^23^. These observations highlight the risk of over-optimistic performance estimates and reinforce the imperative for external validation on more heterogeneous data.

Furthermore, the model’s robustness to simulated domain shifts (Tables 15 and 16) suggests it captures invariant features rather than overfitting to dataset–specific artifacts, supporting its potential for generalizability pending external validation. The sharp drop under Gaussian noise underscores the importance of preprocessing pipelines that include denoising and illumination normalization before images are fed to the model.

However, the performance metrics observed here must be contextualized within the broader landscape of AI in oral health. Recent systematic reviews have highlighted substantial variability in diagnostic accuracy (25%–96%) for AI models in oral lesion assessment, largely dependent on dataset quality, acquisition protocols, and lesion complexity^22^. This variability underscores that results derived from internally curated, single-center cohorts likely represent an upper bound of performance. Without prospective multi-center validation, these metrics may not directly translate to heterogeneous clinical environments where lighting, device variation, and patient demographics differ significantly.

### Interpretability and attention analysis

Grad-CAM visualizations (Supplementary Figs. S5–S8) confirm that MultiDentNet’s attention aligns with diagnostic heuristics by localizing to pathognomonic regions such as carious margins, gingival inflammation, and lesion border irregularities. However, these post-hoc explanations reflect correlation rather than causation and are known to be sensitive to input perturbations^37^. Therefore, they should be viewed as hypothesis-generating tools that support, but do not validate, the model’s clinical reasoning. Visual ambiguity in overlapping pathologies further necessitates clinician-in-the-loop validation and the development of uncertainty-aware modules^36^.

### Comparison with state-of-the-art studies

Recent advances in deep learning (DL) have significantly accelerated automated diagnosis across a broad spectrum of dental and oral pathologies, including caries, periodontitis, gingivitis, oral cancer, and adjacent anatomical lesions. Early efforts primarily leveraged CNN-based architectures for lesion detection and tooth classification: Liu et al.^38^ applied Mask R-CNN for oral disease segmentation, while Chen et al.^39^ and Mahdi et al.^40^ utilized Faster R-CNN variants for tooth recognition, achieving up to 98.2% F1-score. Task-specific classification has also been extensively explored; Askar et al.^41^ employed SqueezeNet for fluorescence abnormality detection (87% accuracy), Alalharith et al.^42^ used ResNet-50 for inflammation classification (77.12%), and Abdalla-Aslan et al.^43^ implemented Cubic SVM for restoration assessment (93.6%). Hybrid and multi-modal pipelines, such as those by Khan et al.^44^, Chang et al.^45^, and Watanabe et al.^46^, combined DL with conventional CAD or panoramic/periapical imaging to stage periodontitis, assess bone loss, and detect complex pathologies like radicular cysts. Beyond tooth-centric tasks, DL has been successfully extended to adjacent structures (e.g., Kuwana et al.^47^ for maxillary sinus lesions) and advanced segmentation frameworks, including automated jaw separation^48^, capsule networks for caries detection^49^, and CNN-Transformer hybrids for precise boundary delineation^50^.

Despite these advancements, most existing frameworks address isolated conditions or rely on single-backbone architectures, limiting their clinical utility for comprehensive, multi-condition screening. To bridge this gap, we propose MultiDentNet, a unified framework that integrates four pre-trained CNN backbones (DenseNet-121, EfficientNetV2-S, ResNet-50, and Inception-V3) enhanced with squeeze-and-excitation (SE) blocks, graph convolutional networks (GCNs), and a multi-task learning strategy. This architecture simultaneously classifies five dental conditions (caries, gingivitis, discoloration, ulcers, hypodontia) alongside oral cancer. On the held-out test set, MultiDentNet achieved 99.68% accuracy for dental conditions and 98.53% for oral cancer, surpassing existing benchmarks (Table 19). These results demonstrate its capacity to jointly capture global contextual dependencies and fine-grained pathological features, underscoring its potential for robust, automated clinical screening. The quantitative comparisons summarized in Table 19 further validate these conclusions.

**Table 19:** Comparison of the proposed MultiDentNet model with state-of-the-art methods on mouth and oral disease classification.

References	Method	Disease	Database	Accuracy
^42^	ResNet-50	Inflamed, non-inflamed	Intraoral images	77.12%
^41^	SqueezeNet	Fluorotic white-spot, other lesions	Self-created database	87.0%
^48^	Fully automatic jaw separation	Dental biometrics	Panoramic radiographs	0.99 (ratio)
^45^	Hybrid DL + CAD	CEJ, cementoenamel, PBL	Panoramic images	91.0%
^49^	Capsule Network	Dental caries	Panoramic images	86.05%
^44^	Faster R-CNN	Caries, ABR, IRR	Dental periapical radiographs	84.76%
^47^	AlexNet, VGG-16, DetectNet	Maxillary incisor region	Periapical radiographs	90%, 72%, 96%
^40^	ResNet-50, ResNet-101	Normal, missing, residual, implants	Periapical radiographs	97.6%, 98.0%
^50^	DenUnet (CNN + Transformer)	Tooth segmentation	DNS dataset	95.4%
Proposed (MultiDentNet)	Weighted ensemble of 4 CNNs + SE + GCN + Multi-task	Caries, Gingivitis, Discoloration, Ulcers, Hypodontia	Multi-condition dental	**99.70%**

ABR, auditory brainstem response; IRR, infusion-related response; CEJ, cementoenamel junction; PBL, periodontal bone loss

## Conclusions

This study presents MultiDentNet, a backbone-diverse ensemble framework integrating squeeze-and-excitation attention, graph convolutional networks for inter-class relational modeling, and multi-task learning to simultaneously screen five common dental conditions and perform binary risk stratification for clinically suspicious oral lesions. Systematic ablation, bootstrapped uncertainty quantification, and domain-shift characterization demonstrate strong internal performance, effective class imbalance handling, and clinically aligned attention. The near-perfect metrics for the dental dataset (accuracy 99.70%, $\kappa =0.996$, AUC = 1.000) reflect an upper bound under controlled acquisition and delineate the operational limits of single-center, image-only models.

Key limitations include: single-dataset evaluation, which constrains generalizability despite rigorous internal validation; a single-label formulation that overlooks common co-occurring conditions; reliance on clinical labeling without exhaustive histopathology, requiring conservative “cancer-suspicious” framing; restriction to 2D RGB images, limiting sub-surface pathology detection; post-hoc interpretability that supports but does not clinically validate model reasoning; and evaluation on aggregate metrics without demographic subgroup analyses, underscoring the need for bias auditing. Additionally, perfect AUC and near-zero loss confirm strong class separation but do not guarantee calibrated probabilities, a known optimistic bias in medical imaging AI, further emphasizing the need for context-aware threshold calibration and external validation.

MultiDentNet is therefore positioned strictly as an adjunctive screening tool for preliminary triage, decision support, and workflow augmentation, especially in resource-limited or tele-dentistry settings. Responsible clinical translation will require prospective multi-center validation, clinician-in-the-loop oversight, context-dependent threshold tuning, and alignment with Software as a Medical Device (SaMD) regulatory frameworks.

Future work will prioritize: (i) multi-center validation with histopathologically verified reference standards; (ii) hierarchical multi-label architectures to capture co-existing conditions; (iii) multimodal fusion with radiographs for sub-surface detection; (iv) privacy-preserving and active learning pipelines for scalable annotation; (v) age-stratified, radiographically confirmed hypodontia models; and (vi) reader studies and clinical trials to assess diagnostic impact, workflow efficiency, and patient outcomes.

In summary, MultiDentNet offers a rigorous, interpretable, and computationally efficient foundation for multi-condition oral screening and lesion risk stratification. By transparently defining performance boundaries, acknowledging dataset and modality constraints, and aligning claims with adjunctive clinical utility, this work provides a reproducible platform for responsible AI translation in oral healthcare. While current results confirm strong technical feasibility, definitive clinical integration awaits external validation, multimodal expansion, and regulatory-compliant trials. MultiDentNet thus represents a meaningful step toward scalable, equitable, and clinician-augmented oral diagnostics, with a clear roadmap for iterative refinement and real-world deployment, ultimately aiming to reduce oral health disparities through accessible, AI-enhanced screening.

## Supplementary Information

Supplementary Information 1

## Data Availability

The datasets can be downloaded from the following links: https://www.kaggle.com/datasets/salmansajid05/oral-diseases, https://www.kaggle.com/datasets/zaidpy/new-oral-cancer/data.

## Code availability

The source code and the experiments presented in this study are openly available in a GitHub repository: https://github.com/Aliyar4061/MultiDentNetV3.
