# Supplementary material for: MultiDentNet: a unified deep learning framework for multi-class dental condition screening and preliminary oral lesion triage
Source: Sci Rep. 2026 May 13;16:21815. doi: 10.1038/s41598-026-52264-6 (PMC13357589; doi:10.1038/s41598-026-52264-6)
Supplement: Supplementary file 1 — Supplementary Information 1 [file 41598_2026_52264_MOESM1_ESM.pdf]

Supplementary Information

1 Supplementary Figures

1.1 Training Dynamics

1.1.1 Dental Conditions

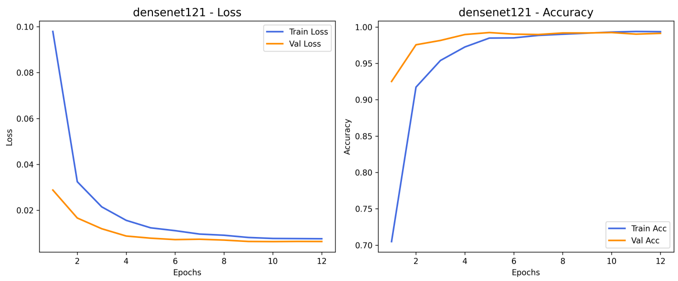

(a) DenseNet121 (Baseline)

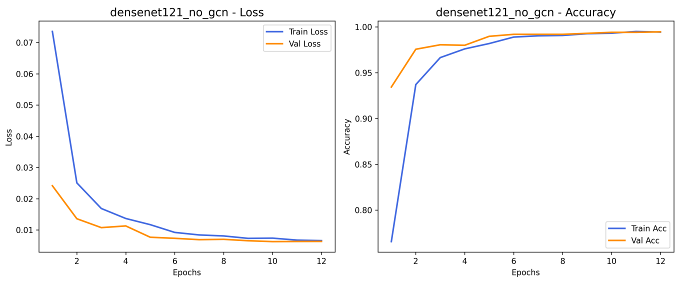

(b) DenseNet121 (No GCN)

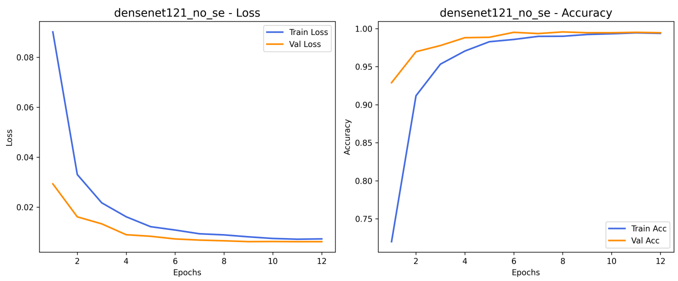

(c) DenseNet121 (No SE)

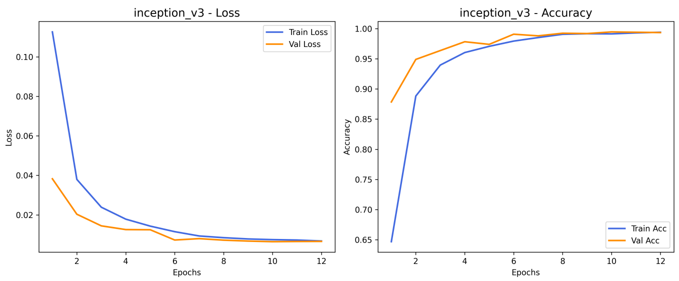

(d) Inception V3

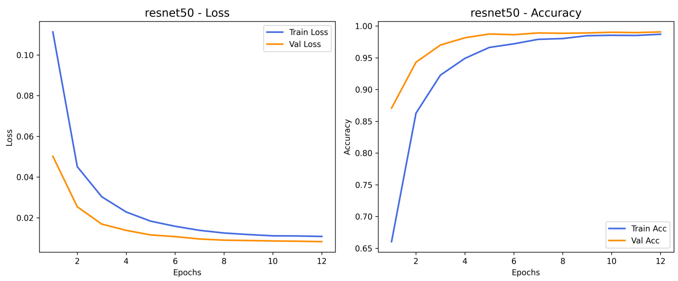

(e) Resnet50

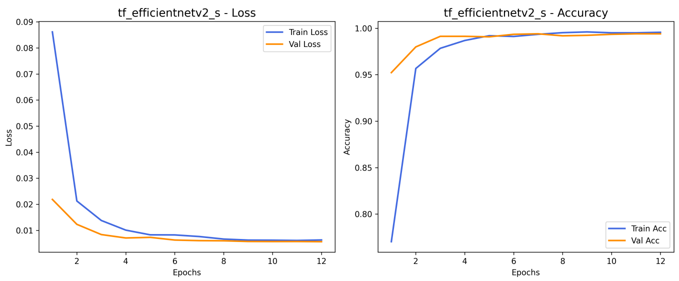

(f) EfficientNetV2-S (Baseline)

Supplementary Fig. S1. Learning curves for dental conditions dataset across model variants

1.1.2 Oral Cancer

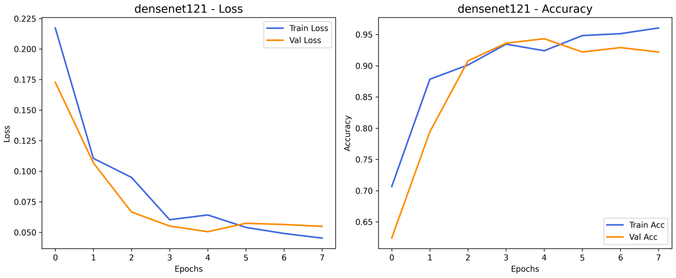

(a) DenseNet121 (Baseline)

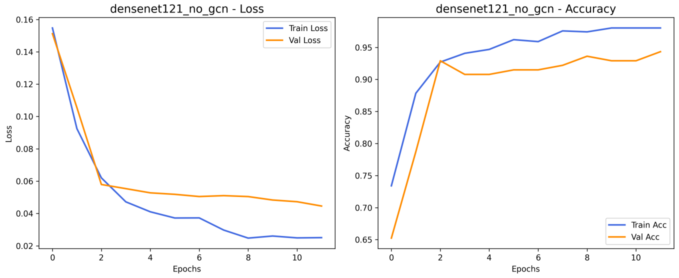

(b) DenseNet121 (No GCN)

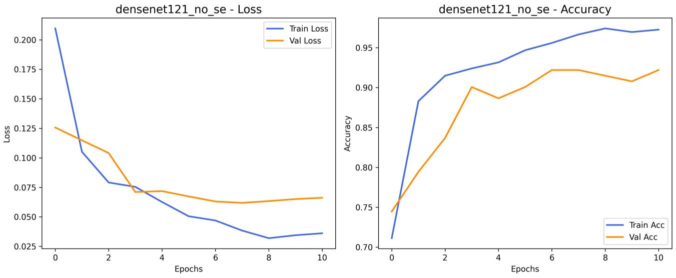

(c) DenseNet121 (No SE)

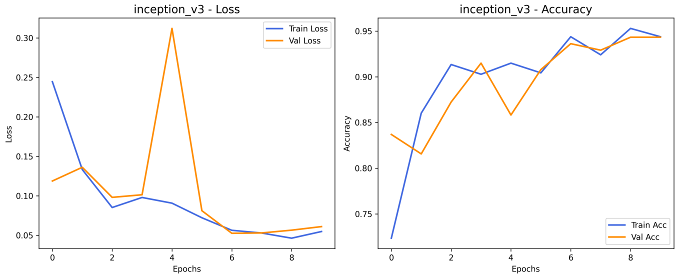

(d) Inception V3

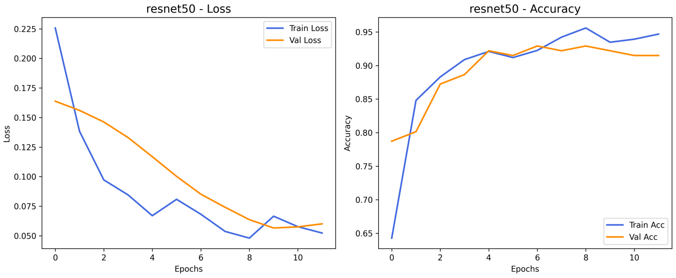

(e) Resnet50

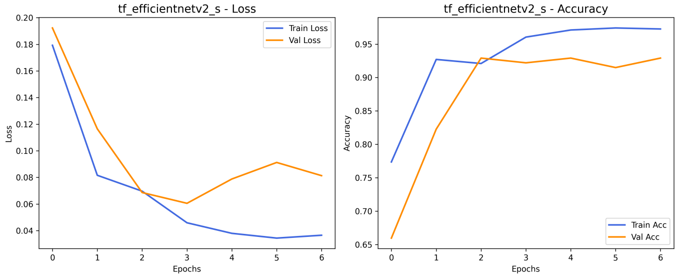

(f) EfficientNetV2-S (Baseline)

Supplementary Fig. S2. Learning curves for oral cancer dataset across model variants

1.2 Confusion Matrices

1.2.1 Dental Conditions

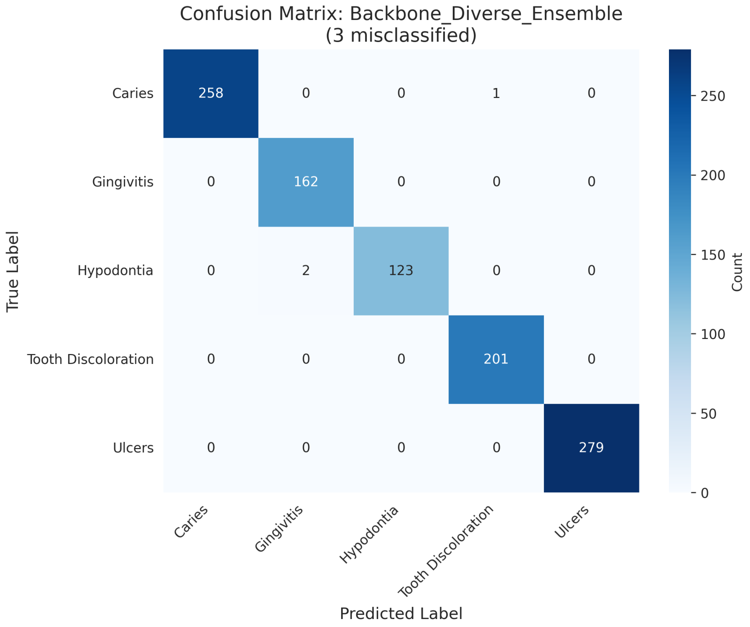

(a) Backbone diverse ensemble

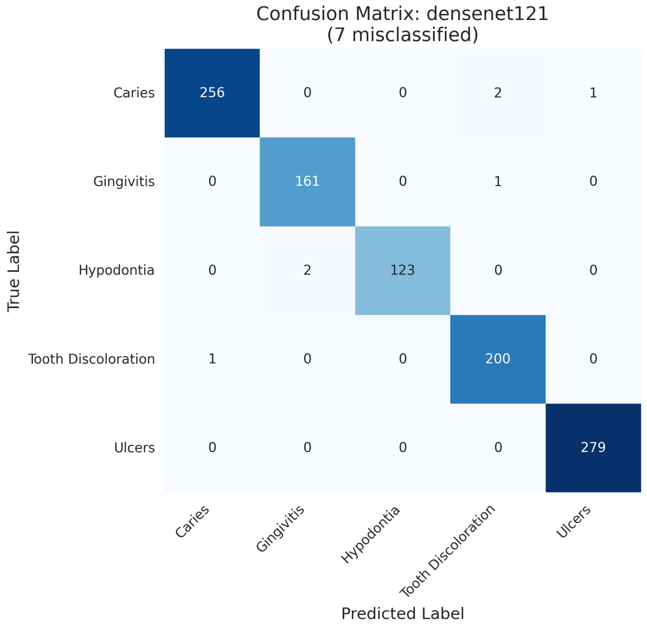

(b) DenseNet121 (Baseline)

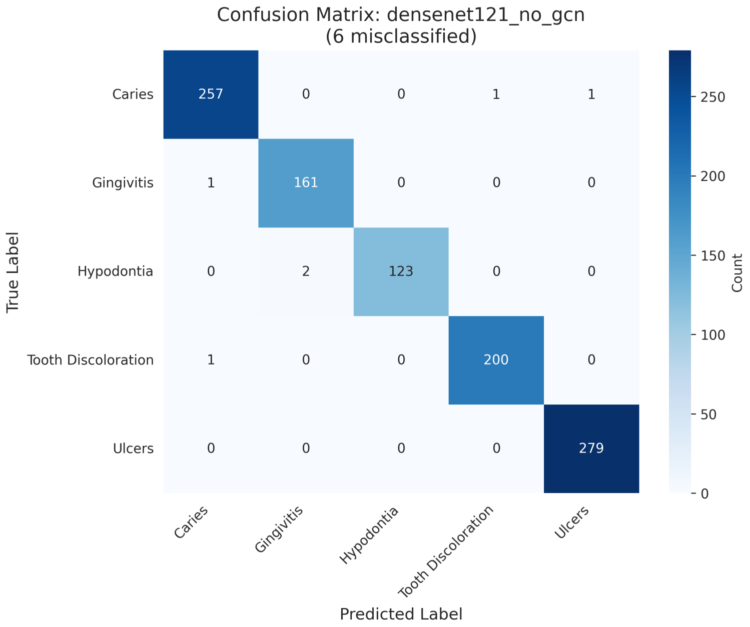

(c) DenseNet121 (No GCN)

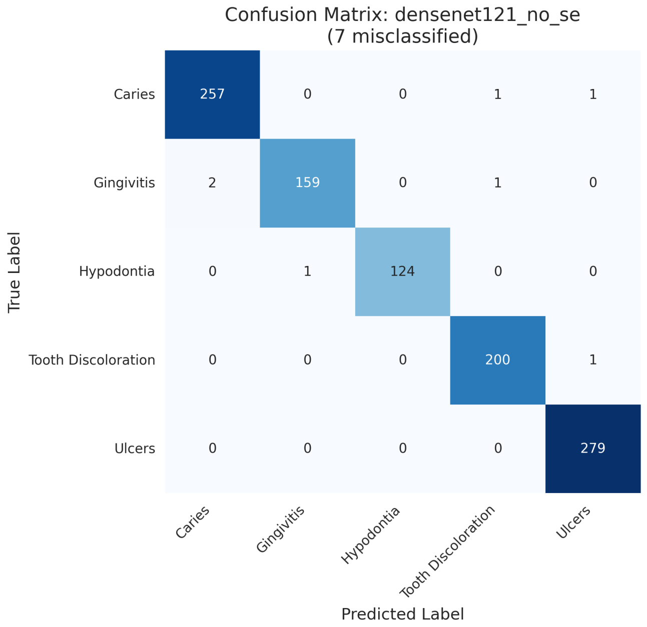

(d) DenseNet121 (No SE)

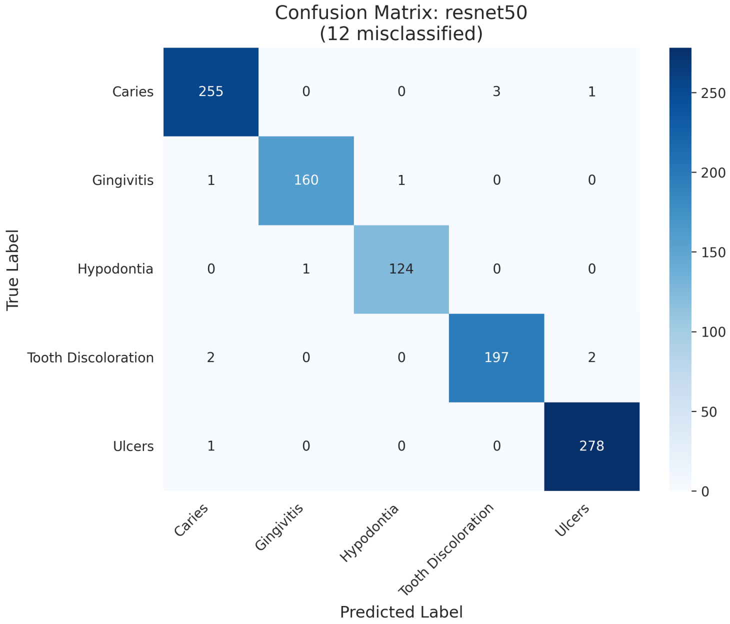

(e) ResNet50

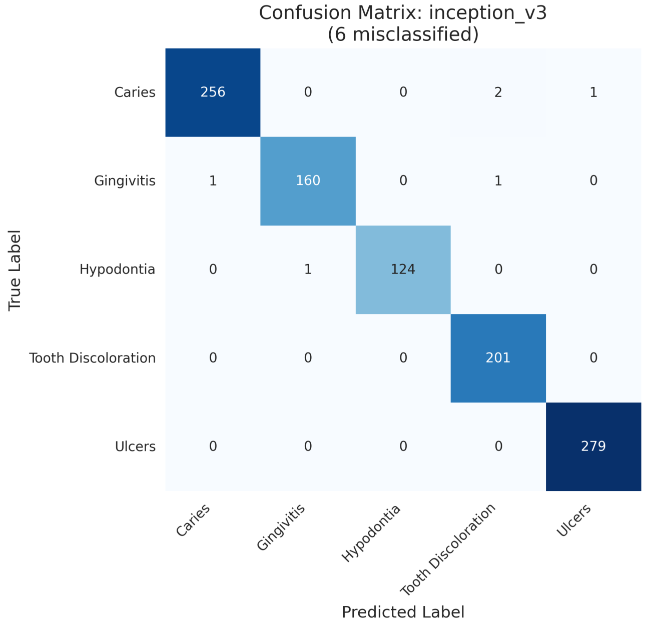

(f) Inception V3

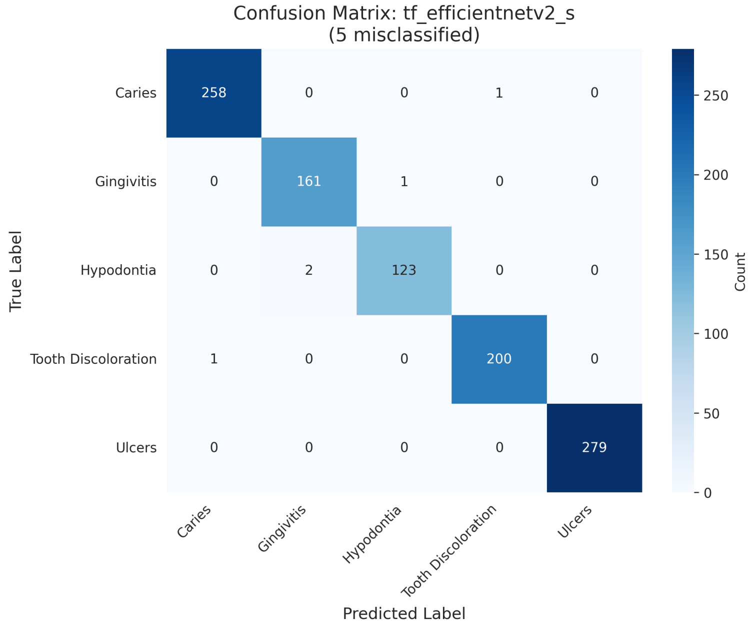

(g) EfficientNetV2-S

Supplementary Fig. S3. Confusion matrices related to dental dataset with respect to different models

1.2.2 Oral Cancer

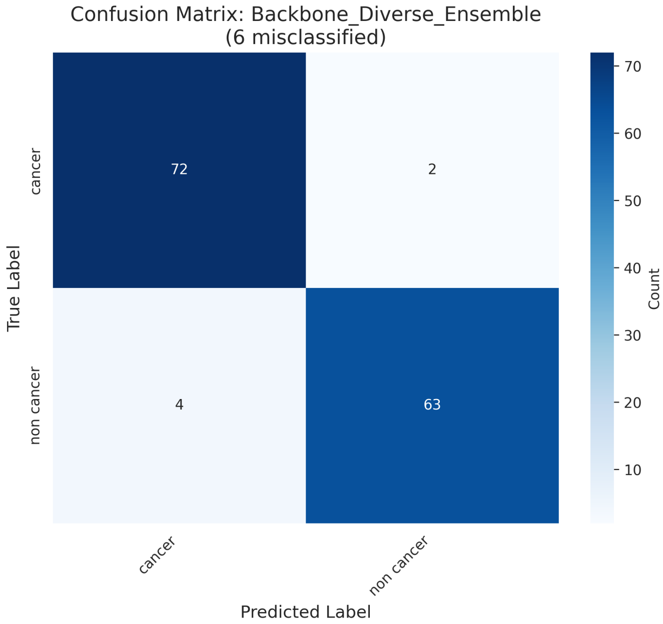

(a) Backbone diverse ensemble

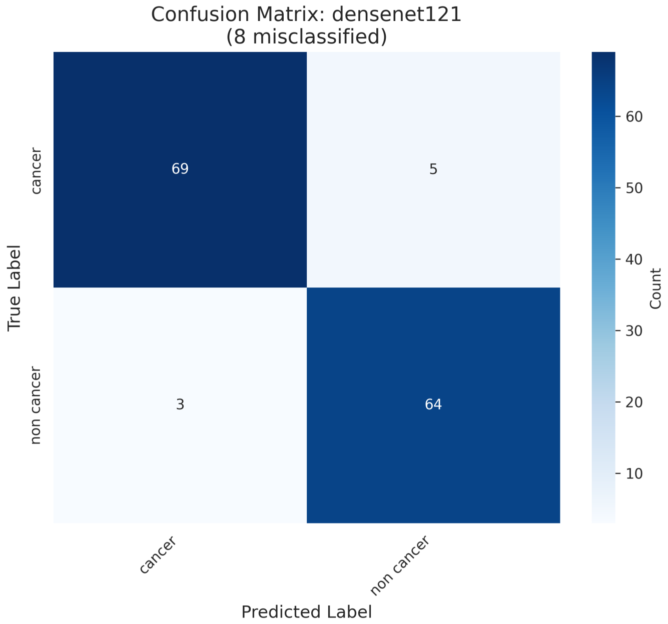

(b) DenseNet121 (Baseline)

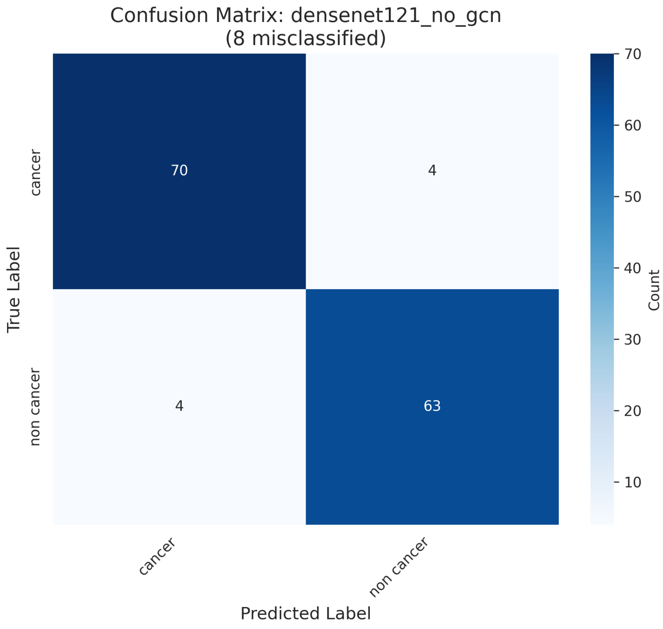

(c) DenseNet121 (No GCN)

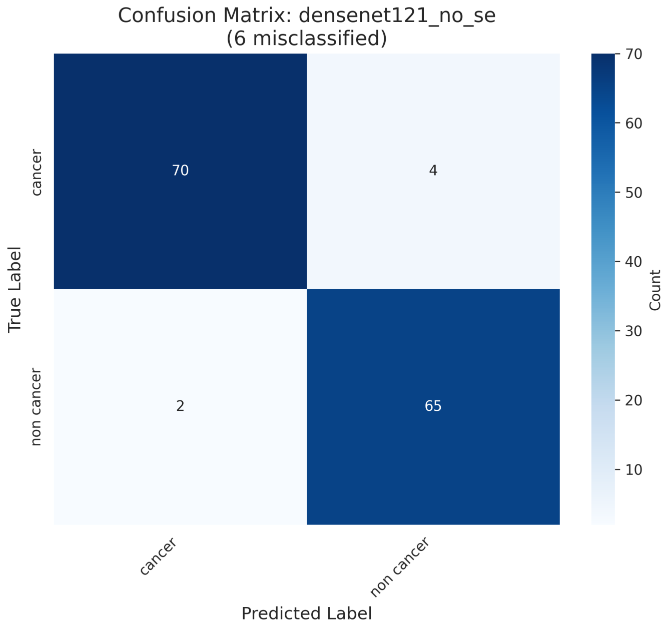

(d) DenseNet121 (No SE)

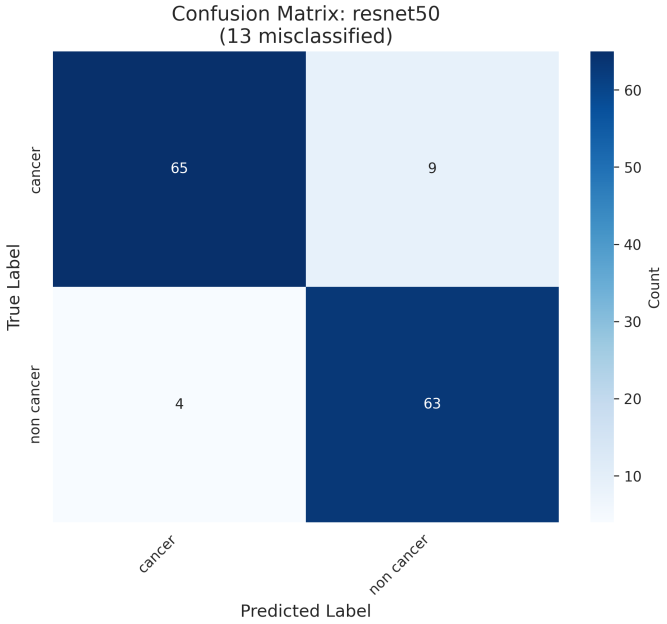

(e) ResNet50

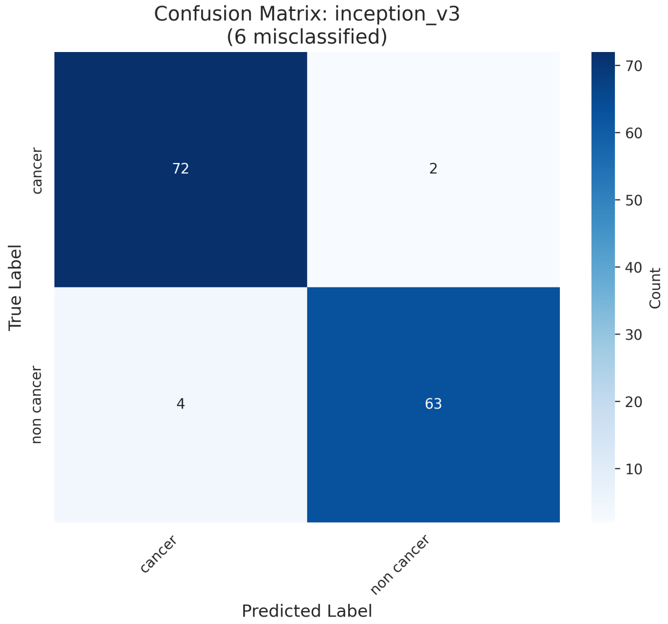

(f) Inception V3

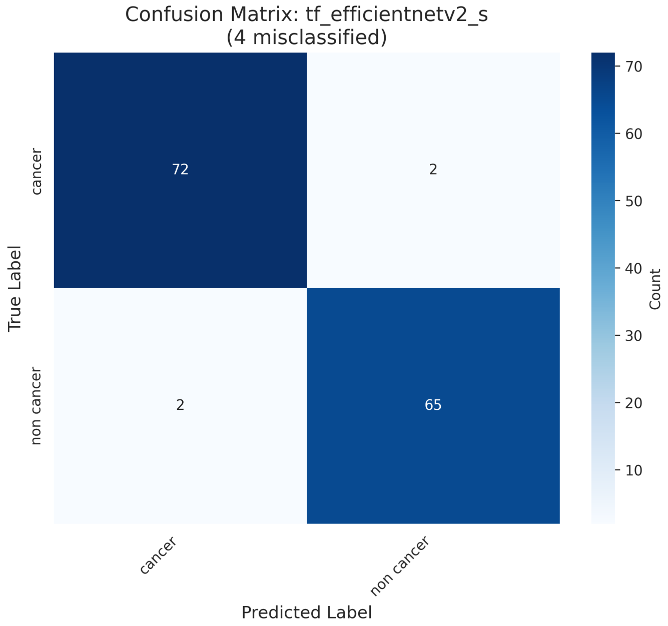

(g) EfficientNetV2-S

Supplementary Fig. S4. Confusion matrices related to oral dataset with respect to different models

2 Supplementary Classification Examples

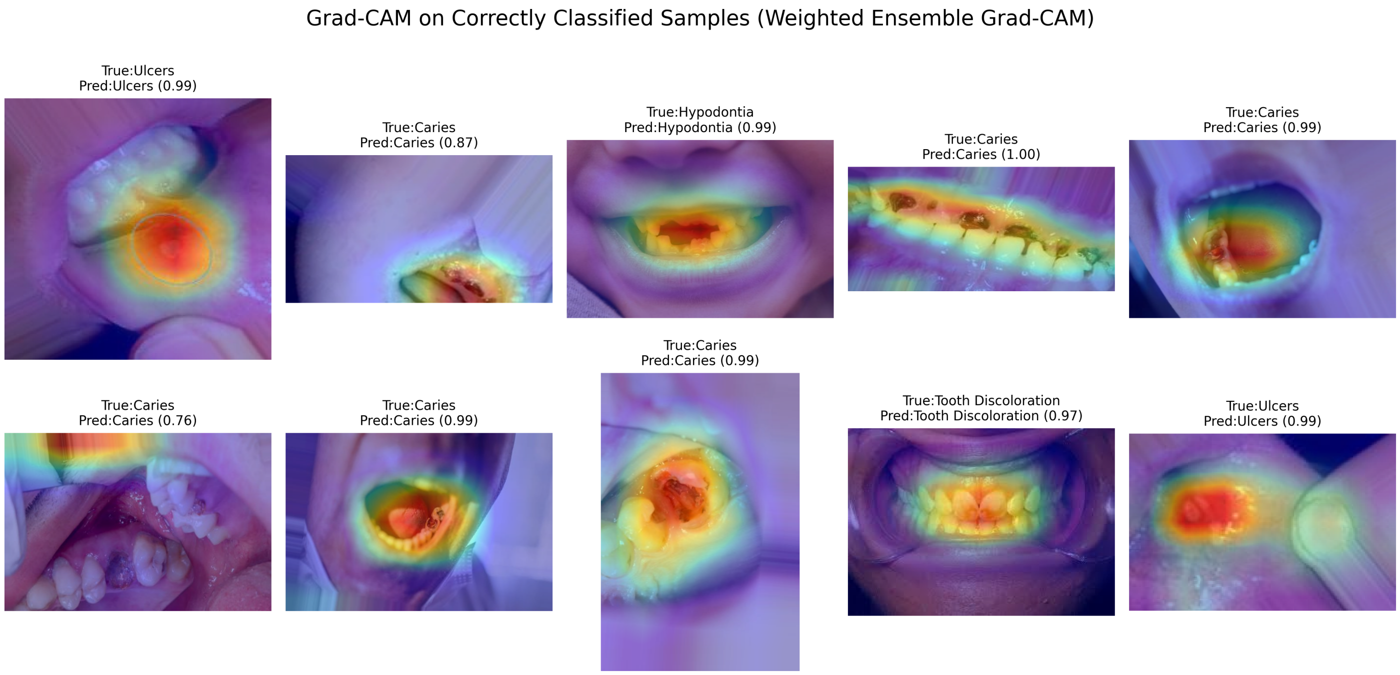

Supplementary Fig. S5. Grad-CAM on correctly classified samples of dental dataset

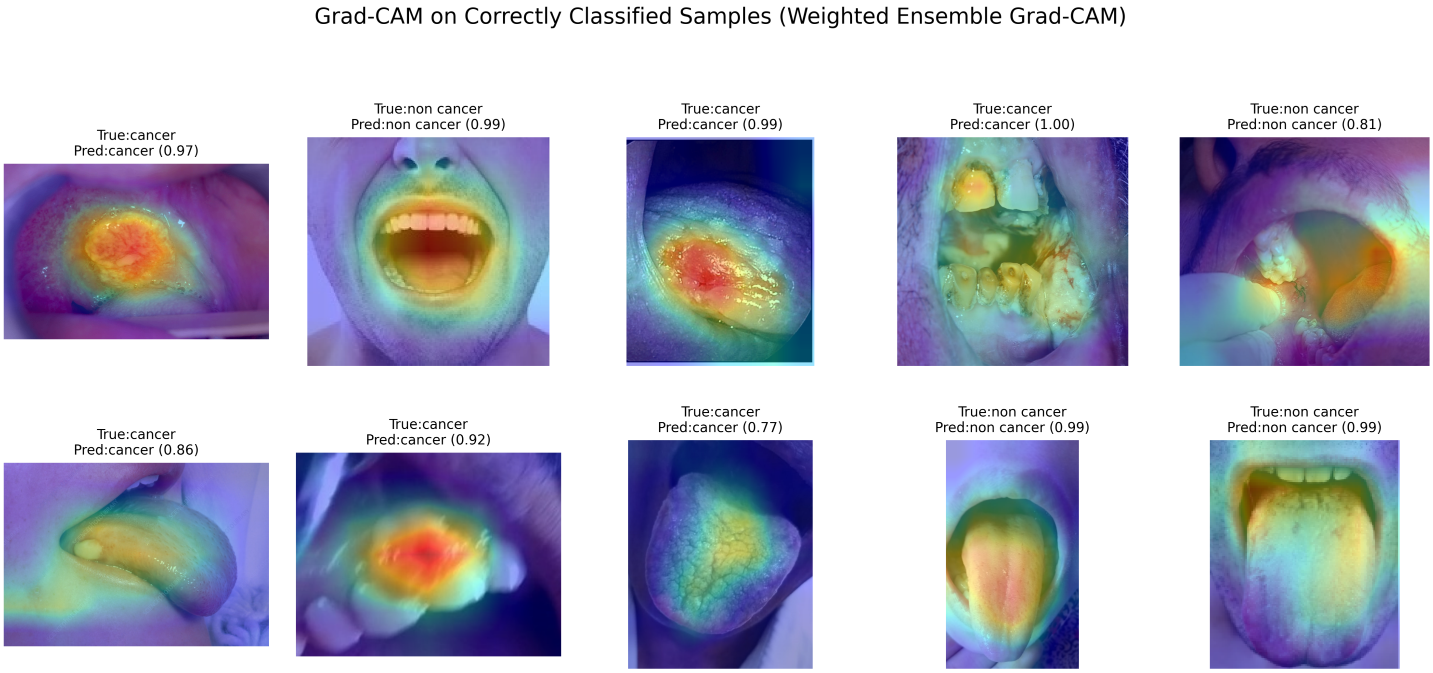

Supplementary Fig. S6. Grad-CAM on correctly classified samples of oral dataset

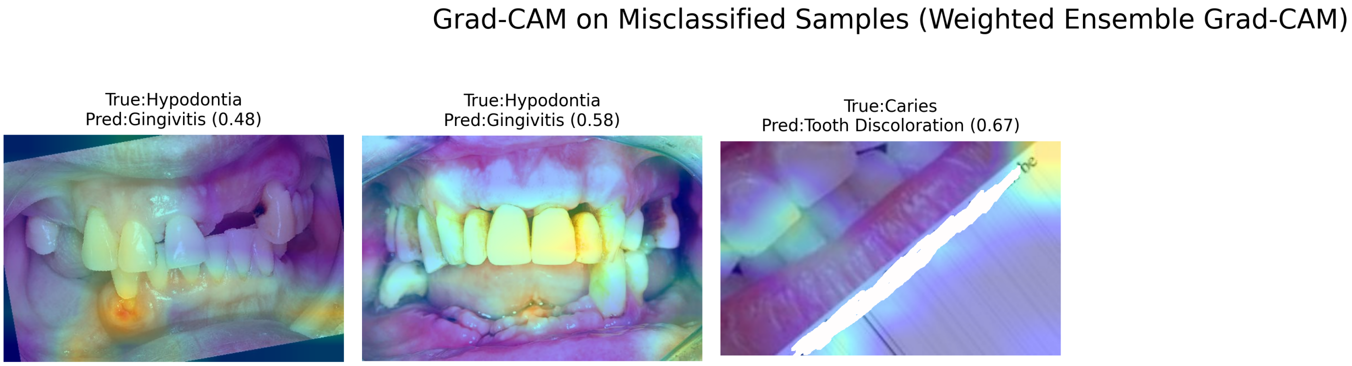

Supplementary Fig. S7. Grad-CAM on misclassified samples of dental dataset

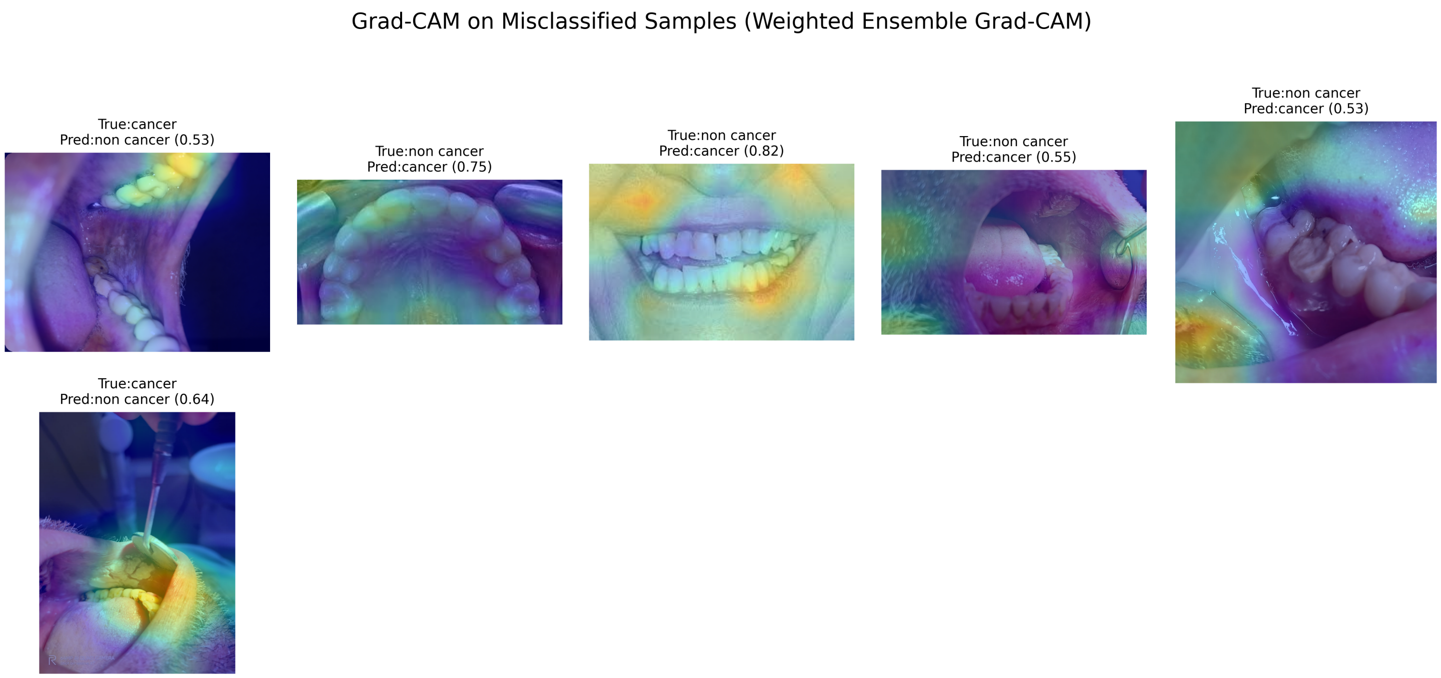

Supplementary Fig. S8. Grad-CAM on misclassified samples of oral dataset
